# Can bio-H_2_ compete with bio-CH_4_–unequal chances of anaerobic digestion metabolic pathways

**DOI:** 10.3389/fmicb.2026.1881665

**Published:** 2026-07-28

**Authors:** Anna Sikora, Anna Detman-Ignatowska

**Affiliations:** Laboratory of White Biotechnology, Institute of Biochemistry and Biophysics PAS, Warsaw, Poland

**Keywords:** anaerobic digestion, biohydrogen, biomethane, dark fermentation, metabolic pathways, microorganisms

## Abstract

Biogas, a mixture of biomethane (bio-CH_4_) and carbon dioxide (CO_2_), is the product of methanogenesis, the final stage of anaerobic digestion (AD). Biohydrogen (bio-H_2_), on the other hand, is produced during the acidogenesis stage through dark fermentation (DF) processes and the transformation of intermediates from other fermentation types, such as the conversion of lactate and acetate into butyrate. Bio-H_2_ and CO_2_ together form fermentation gas, whose volume and bio-H_2_ content depend on the dominant type of acid fermentation. Understanding the complexity of metabolic pathways and the interactions between microorganisms at each stage of AD permits process optimization in two-stage systems, facilitating the production of both bio-H_2_ and bio-CH_4_. Theoretical calculations indicate that separate recovery of bio-H_2_ and bio-CH_4_ through a two-stage AD process yields a higher energy output from the degraded substrate. However, DF is characterized by limited bio-H_2_ yields due to inherent metabolic constraints and competition among microbial pathways. In contrast, acetogenic pathways consistently generate substrates for methanogenesis. Paradoxically, analyses based on simple substrates such as glucose facilitate a deeper understanding of metabolic pathways and chemical reactions in bacterial cells. Such knowledge is essential for the rational design and critical evaluation of integrated multiproduct AD systems, supporting mindful resource management and sustainable development. This review focuses on (i) the biological limitations of bio-H₂ generation during acidogenesis; (ii) physiological constraints that keep bio-H₂ technologies at the research and development stage, compared with the commercially established biogas technologies; and (iii) prospects for bio-H_2_ recovery within integrated multiproduct AD systems.

## 1 Gaseous biofuels

### 1.1 Biohydrogen and biomethane

The term gaseous biofuels refers to bio-H_2_ (as an energy carrier) and bio-CH_4_, both of which are produced by the metabolic activity of microorganisms during anaerobic digestion (AD) of organic compounds. The final product of this process is biogas, a mixture of bio-CH_4_ (50–80%) and CO_2_ (20–50%), plus minor components such as hydrogen sulfide, molecular nitrogen, oxygen, carbon monoxide, ammonia and siloxanes (Thauer et al., 2008; Sieber et al., 2012; Chojnacka et al., 2015; Mao et al., 2015; Bharathiraja et al., 2016). Bio-H_2_ is a gaseous product of acidogenesis and therefore serves as an intermediate in the anaerobic breakdown of biomass. The content of bio-H_2_ in the fermentation gas following acidogenesis ranges from 30–50%, with the remaining components being CO_2_ (50–70%) and trace amounts of other gases, similarly to biogas (Chojnacka et al., 2011; Bharathiraja et al., 2016; Sen et al., 2016; Detman et al., 2017; Castelló et al., 2020; Ahmad et al., 2024).

### 1.2 Anaerobic digestion as a source of gaseous biofuels

AD occurs naturally in oxygen-poor environments with low redox potential and plays a crucial role in the carbon cycle and energy flow within ecosystems. AD is the fundamental process enabling the operation of biogas plants, and also occurs in landfills and wastewater treatment plants, where biogas can be recovered (Thauer et al., 2008; Mao et al., 2015; Sikora et al., 2017; Kougias and Angelidaki, 2018; Centurion et al., 2024).

**Figure 1:**
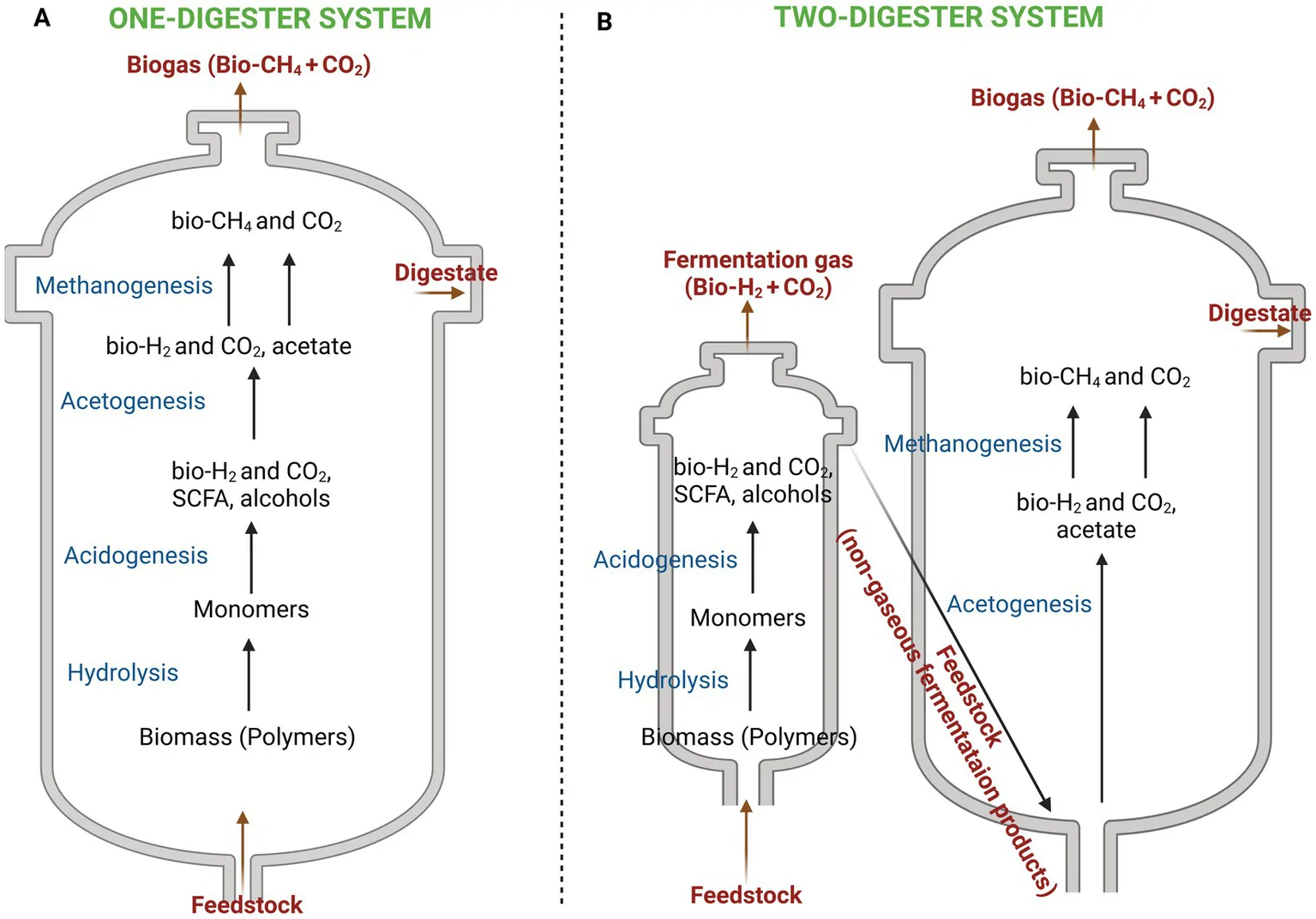
Stages of AD and two systems for carrying out this process: **(A)** In a single-digester system all four stages occur simultaneously. **(B)** In a two-digester system hydrolysis and acidogenesis take place in the first bioreactor, while acetogenesis and methanogenesis occur in the second bioreactor. This separation permits recovery of bio-H₂ during the acidogenic step.

The complex process of anaerobic biomass degradation is divided into four main stages (Figure 1). The first is hydrolysis, during which polymeric organic compounds are broken down into monomers. The second stage is acidogenesis, producing short-chain fatty acids (SCFAs), alcohols and fermentation gas (a mixture of bio-H_2_ and CO_2_). In the third stage, acetogenesis, non-gaseous products of acid fermentation are oxidized to generate bio-H_2_, CO_2_ and acetate, which are the substrates for methanogenic archaea in the fourth stage, methanogenesis. The final two stages, acetogenesis and methanogenesis, are closely linked through syntrophic relationships between acetogenic bacteria and methanogens.

Bio-CH_4_ can be produced from acetate (acetotrophic pathway), from CO_2_ and bio-H_2_ (hydrogenotrophic pathway), or *via* the reduction of methyl groups (methylotrophic pathway) (Sieber et al., 2012; Schink and Stams, 2013; Sikora et al., 2017; Detman et al., 2018b, 2021a; Nguyen et al., 2019; Ghiotto et al., 2024). When there is no temporal and spatial separation between acidogenesis and acetogenesis/methanogenesis, the final product of AD is a mixture of bio-CH_4_ and CO_2_. Where a separation between these stages occurs, acidogenesis yields fermentation gas containing bio-H_2_ and CO_2_, as well as non-gaseous fermentation products (Figure 1) (Chojnacka et al., 2011; Schievano et al., 2014; Detman et al., 2017, 2021c; Sikora et al., 2017; Mari et al., 2020; Sikora, 2022; Eggers et al., 2024; Devens et al., 2025).

This review focuses on the metabolic pathways and microbial interactions involved in acidogenesis, acetogenesis, and methanogenesis, which form the biological basis of multiproduct technologies based on anaerobic biomass digestion, including biohydrogen production *via* dark fermentation (DF). A thorough understanding of these pathways and interactions is crucial for the development and optimization of such technologies.

### 1.3 Theoretical energy yield from gaseous biofuels produced via AD

If we approach the issue of AD purely theoretically, treating biomass as a glucose molecule, the acidogenesis process generates bio-H_2_, CO_2_ and acetate in the following reaction:


$$C_{6}H_{12}O_{6}+2H_{2}O\to 2{\mathrm{CH}}_{3}\text{COOH}+2{\mathrm{CO}}_{2}+4H_{2}$$

In this scenario, acetogenesis is not required since there are ready substrates for methanogenesis, which produces bio-CH_4_ from acetate:


$$2{\mathrm{CH}}_{3}\text{COOH}\to 2{\mathrm{CH}}_{4}+2{\mathrm{CO}}_{2}$$

and from bio-H_2_ and CO_2_:


$$2{\mathrm{CO}}_{2}+4H_{2}\to {\mathrm{CH}}_{4}+2H_{2}O+{\mathrm{CO}}_{2}$$

The overall methanogenic reaction looks like this:


$$C_{6}H_{12}O_{6}+2H_{2}O\to 2{\mathrm{CH}}_{4}+3{\mathrm{CO}}_{2}+2H_{2}O$$

or in a simplified form:


$$C_{6}H_{12}O_{6}\to 3{\mathrm{CH}}_{4}+3{\mathrm{CO}}_{2}$$

When the process is carried out in two separate stages, i.e., acidogenesis occurs independently, one mole of glucose yields 4 moles of bio-H_2_ and 2 moles of bio-CH_4_. When all stages proceed simultaneously, 3 moles of bio-CH_4_ are produced.

Taking into account the heat of combustion of both gases (891.6 kJ/mol for CH_4_ and 285.8 kJ/mol for H_2_), the energy yield from the system producing 2 moles of bio-CH_4_ and 4 moles of bio-H_2_ is 2 × 890 + 4 × 286 = 1780 + 1,144 = 2,924 kJ. In the variant generating 3 moles of bio-CH_4_, the energy obtained is 3 × 890 = 2,670 kJ. This demonstrates that a multi-stage AD process with bio-H_2_ recovery can yield more energy than a simultaneous and complete digestion. These theoretical considerations are supported by practical experimental approaches for a range of substrates (Schievano et al., 2012, 2014; Mari et al., 2020; Sołowski et al., 2020; Bertasini et al., 2024; Corrêa et al., 2025; Özmıhçı et al., 2025).

Although thermodynamic analyses based on simplified substrates such as glucose provide valuable insights into the energetic feasibility of bio-H_2_-producing pathways, they represent highly idealized systems. Even under these simplified conditions, DF is characterized by limited bio-H_2_ yields due to inherent metabolic constraints and competition among microbial pathways. When transitioning to complex industrial feedstocks, additional limitations arising from substrate heterogeneity, microbial community dynamics, and competing metabolic routes further reduce the feasibility of maximizing bio-H_2_ production as a standalone process. Therefore, these thermodynamic simplifications reinforce, rather than weaken, the central argument of this review: bio-H_2_ should be regarded as an intermediate energy carrier recovered within an integrated multiproduct AD technology, rather than as the sole target product.

## 2 Challenges and limitations of bio-H_2_ production during acidogenesis arising from the biological nature of the processes

### 2.1 Bio-H_2_-yielding fermentations

**Figure 2:**
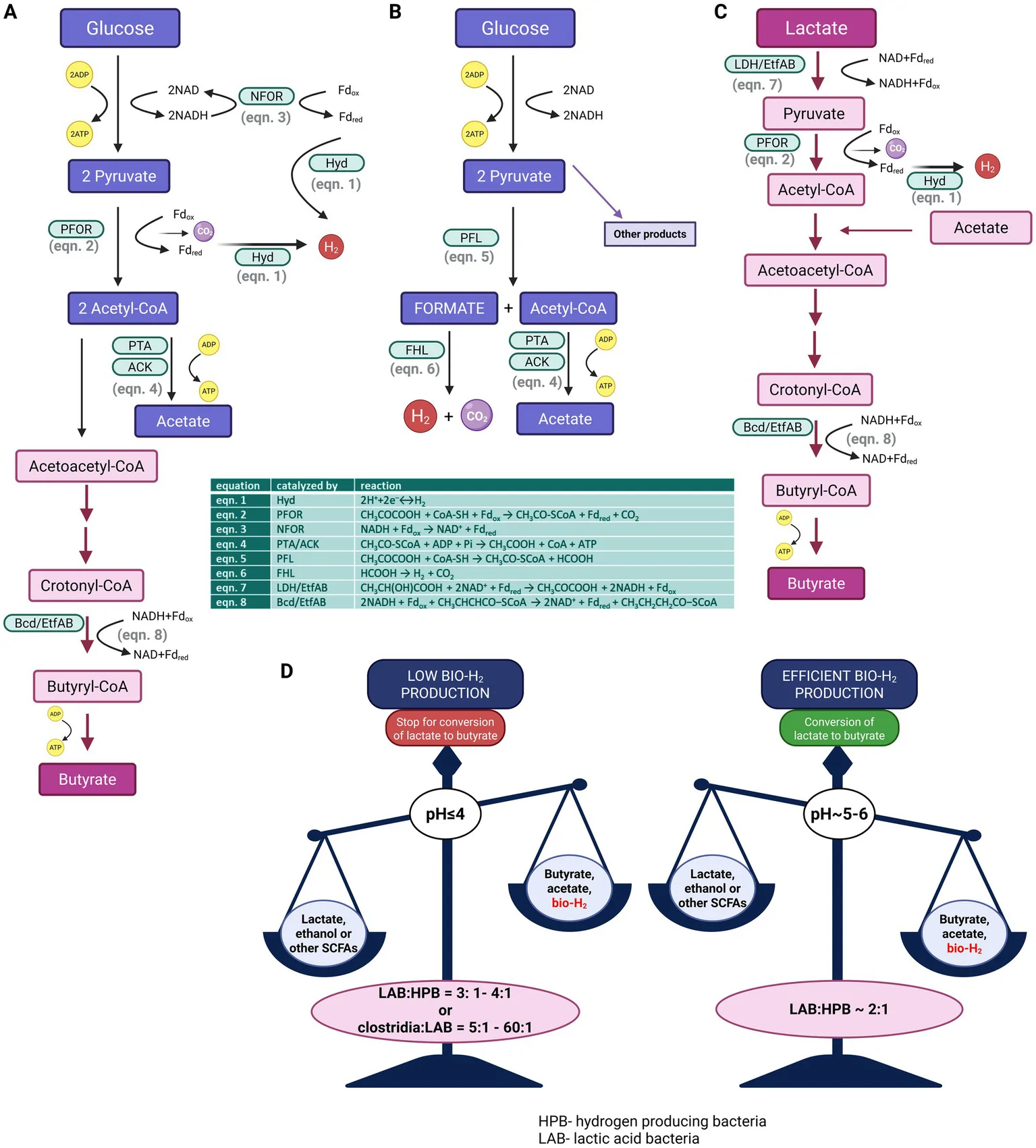
Bio-H₂-yielding metabolic pathways in bacterial cells: **(A)** *Clostridium*-type fermentation – red arrows indicate the reactions leading to the theoretical bio-H_2_ yield of 4 moles of H₂ per mole of glucose, according to the reaction C₆H₁₂O₆ + 2H₂O → 2CH₃COOH + 2CO₂ + 4H₂, where acetate is the only non-gaseous product. Green arrows show the pathway leading to butyrate formation, with a bio-H_2_ yield of 2 moles of H₂ per mole of glucose, according to the reaction C₆H₁₂O₆ + 2H₂O → 2H₂ + 2CO₂ + CH₃CH₂CH₂COOH. **(B)** *Enterobacteriaceae*-type fermentation. **(C)** Conversion of lactate and acetate to butyrate – note the similarities to *Clostridium*-type fermentation, where butyrate is also formed. **(D)** Microbial balance in bio-H₂-producing microbial communities based on (Detman et al., 2019, 2021c). The optimal conditions for bio-H₂ production are determined by technical aspects and operational parameters such as hydraulic retention time (HRT), temperature, pH, feedstock composition, bioreactor construction and material, packing material, and methods of substrate feeding and distribution. Hyd, hydrogenase; PFOR, pyruvate: ferredoxin oxidoreductase; NFOR, NADH: ferredoxin oxidoreductase; PTA, phosphotransacetylase; ACK, acetate kinase; PFL, pyruvate formate lyase; FHL, formate hydrogenlyase; LDH/EtfAB, FAD-dependent lactate dehydrogenase/electron transfer flavoprotein complex; Bcd/EtfAB, butyryl-CoA dehydrogenase/EtfAB complex.

In the theoretical scenario described above, glucose is the sole substrate for one type of acid fermentation, yielding acetic acid, bio-H_2_ and CO_2_ (*Clostridium*-type fermentation described below). However, in practice, even if glucose is the only substrate, multiple fermentation types can occur at the acidogenesis stage. Glucose and other monosaccharides are metabolized primarily through glycolytic fermentation. The intermediate product of these transformations is pyruvate, which, depending on the type of fermentation, is converted into ethanol (alcoholic fermentation), lactic acid (lactic fermentation), or oxaloacetate (propionic fermentation). In these types of fermentation the sole gaseous product is CO_2_, with no bio-H_2_ generation. Fermentations that produce bio-H_2_ are classified as DF and two main types are recognized: *Clostridium*-type (Figure 2A) and *Enterobacteriaceae*-type (Figure 2B) (Hallenbeck, 2005, 2009; Kraemer and Bagley, 2007; Das and Veziroglu, 2008; Lee et al., 2011; Ramprakash et al., 2022; D’ Silva et al., 2023; Ahmad et al., 2024). In *Clostridium*-type fermentation, pyruvate is converted into acetyl-CoA and simultaneously ferredoxin is reduced and bio-H_2_ is released through the action of [Fe-Fe] and [Ni-Fe] hydrogenases (equation 1, Figure 2A) (Shima et al., 2025). Formation of acetyl-CoA is catalysed by pyruvate:ferredoxin oxidoreductase (PFOR) in the presence of ferredoxin (Fd) (equation 2, Figure 2A), while NADH:ferredoxin oxidoreductase (NFOR) reduces ferredoxin in a reaction with NADH (equation 3, Figure 2A). The latter reaction relies on electron bifurcation, coupling exergonic NADH oxidation with endergonic ferredoxin reduction in a single electron transfer process (Mostafa et al., 2022). The formation of acetate from acetyl-CoA, catalyzed by phosphotransacetylase (PTA) and acetate kinase (ACK), is an ATP-generating reaction for the bacterial cell (equation 4 in Figure 2A). The main reason why more bio-H_2_ cannot be obtained in fermentations is that the main goal of these processes is to produce energy for the bacterial cell. Therefore, the theoretical maximum bio-H_2_ yield during clostridial-type fermentation decreases from 4 moles of bio-H_2_ per mole of substrate to 2 when glucose is converted to butyrate according to the reaction:


$$C_{6}H_{12}O_{6}+2H_{2}O\to 2H_{2}+2{\mathrm{CO}}_{2}+{\mathrm{CH}}_{3}{\mathrm{CH}}_{2}{\mathrm{CH}}_{2}\text{COOH}$$

The formation of other non-gaseous fermentation products (solventogenesis) causes a further decrease in bio-H_2_ yield (Hallenbeck, 2005; Kraemer and Bagley, 2007; Lee et al., 2011; Ramprakash et al., 2022).

In *Enterobacteriaceae*-type fermentation (mixed acid-fermentation or formate fermentation), pyruvate is converted into formate and acetyl-CoA by pyruvate formate lyase (PFL) (equation 5, Figure 2B). The formate is converted into bio-H_2_ and CO_2_ by formate hydrogenlyase (FHL) (equation 6, Figure 2B). In the *Enterobacter*-subtype of *Enterobacteriaceae*-type fermentation, bio-H_2_ is also generated through oxidation of NADH by NFOR in reactions similar to those described for the clostridial-type fermentation. However, the theoretical bio-H_2_ yields from *Enterobacteriaceae*-type fermentation are lower than those reported for clostridial-type fermentation (Hallenbeck, 2005; Kraemer and Bagley, 2007; Das and Veziroglu, 2008; Cha et al., 2025; Fan et al., 2025).

### 2.2 Other bio-H_2_-yielding pathways during acidogenesis

The transformation of products from other fermentation pathways during acidogenesis also contributes to bio-H_2_ formation. Such processes include the conversion of lactate and acetate to butyrate, with the release of H_2_, as reported for, e.g., *Clostridium acetobutylicum* (Diez-Gonzalez et al., 1995), *C. diolis* (Matsumoto and Nishimura, 2007), *C. tyrobutyricum* (Jo et al., 2008), *C. butyricum* (Detman et al., 2019) and *Butyribacterium methylotrophicum* (Shen et al., 1996). This phenomenon is analogous to the cross-feeding of lactate described in the gut microbiome, resulting from nutritional symbiotic relationships between lactic acid bacteria (LAB) and butyrate producers (Duncan et al., 2004; Muñoz-Tamayo et al., 2011). The end products from LAB fermentation are used as substrates by butyrate producers, even though each group can live independently of the other. Studies of the acidogenic phase of AD using various substrates and experimental approaches have clearly demonstrated the occurrence of cross-feeding interactions in DF bioreactors, which enhance biohydrogen (bio-H₂) production (Matsumoto and Nishimura, 2007; Baghchehsaraee et al., 2009; Chojnacka et al., 2011; Kim et al., 2012; Sikora et al., 2013; Fuess et al., 2018, 2019; García-Depraect and León-Becerril, 2018; Detman et al., 2019, 2021c, 2021b; García-Depraect et al., 2019a, 2019b, 2019c, 2020, 2021a, 2021b, 2022; Alves De Oliveira et al., 2020; Martínez-Mendoza et al., 2022; Sikora, 2022; Regueira-Marcos et al., 2023).

The key reaction in the conversion of lactate to butyrate is the initial step mediated by an electron-bifurcating enzyme complex comprised of FAD-dependent lactate dehydrogenase (LDH) plus an electron transfer flavoprotein (EtfAB) (Figure 2C). This catalyzes the endergonic oxidation of lactate using NAD^+^ as the oxidant, which is accompanied by the simultaneous oxidation of reduced ferredoxin (equation 7, Figure 2C) (Weghoff et al., 2015).

The subsequent steps follow the same pathway as clostridial-type fermentation, as described in Section 2.1. The formation of butyrate involves the initial condensation of two molecules of acetyl-CoA to produce acetoacetyl-CoA, which requires an additional source of acetate. The key step is the reduction of crotonyl-CoA to butyryl-CoA, again involving an electron bifurcation mechanism. The critical enzyme, a butyryl-CoA dehydrogenase/EtfAB complex (Bcd/EtfAB), catalyzes the endergonic reduction of ferredoxin with NADH coupled to the exergonic reduction of crotonyl-CoA with NADH (equation 8, Figure 2C) (Li et al., 2008).

The conversion of lactate and acetate to butyrate by *C. butyricum* described in Figure 2C is an updated scheme for this pathway, involving flavin-based electron bifurcation, proposed by Detman et al. (2019) (Detman et al., 2019). An analysis of genes encoding EtfAB complexes in bacteria capable of converting lactate and acetate to butyrate (eg., *C. acetobutylicum* and *C. diolis*), butyrate-producers only (eg., *Roseburia intestinalis* and *Eubacterium rectale*) and the lactate oxidizer *Acetobacterium woodii*, revealed that the Etf complexes involved in lactate oxidation and butyrate synthesis form distinct phylogenetic clusters (Detman et al., 2019).

### 2.3 Microbial balance in DF communities

DF bacterial communities represent a distinct microbiome in which LAB usually form a significant component. LAB are generally regarded as inhibitory to DF processes when they become dominant within the microbial community. Analyses of low-performing bio-H₂-producing bioreactors consistently correlate the reduced bio-H_2_ yields with LAB-dominated communities. This effect has been attributed to (i) competition for substrates, (ii) a shift in the dominant fermentation pathway from DF to lactic acid fermentation, (iii) excessive acidification, and (iv) the production of bacteriocins (Hallenbeck, 2009; Bundhoo and Mohee, 2016; Elbeshbishy et al., 2017; Castelló et al., 2020).

Analysis of microbial communities in high bio-H_2_-producing DF bioreactors indicates that the optimal ratio between bio-H_2_-producing bacteria and LAB falls within the range of 1:2 to 3:1, with a median of 3:2. An overabundance of either group results in a significant reduction in bio-H₂ production and the overproduction of non-gaseous fermentation products, such as lactic acid and other SCFAs, indicating a metabolic shift toward solventogenesis. A key challenge appears to be determining the specific balance between bio-H₂-producing bacteria and LAB, which governs the conversion of lactate and acetate into butyrate (Figure 2D) and is therefore essential for stable bio-H₂ production. This balance is influenced by the source of the inoculum and its long-term selection, as well as by operating conditions, including bioreactor design, packing material, hydraulic retention time (HRT), and substrate concentration (Hung et al., 2007; Yang et al., 2007; Etchebehere et al., 2016; García-Depraect et al., 2017, 2019c, 2019b, 2021a; Fuess et al., 2018; García-Depraect and León-Becerril, 2018; Palomo-Briones et al., 2018; Diaz-Cruces et al., 2020; Detman et al., 2021b, 2021c).

It was also recognized that the production of bio-H_2_, SCFAs and alcohols during acidogenesis depends on the redox potential and electron transfer between enzymatic complexes, which channel NADH towards reactions leading to bio-H_2_ formation or solventogenesis (Hallenbeck, 2005; Bundhoo and Mohee, 2016; Elbeshbishy et al., 2017; Atilano-Camino et al., 2020). These findings indicate the requirement for suitable redox mediators that control and stabilize the redox potential and electron transfer within DF bioreactors (Yang and Wang, 2018). Biochar is a promising stabilizer of dark-fermentation processes (Bu et al., 2021; Lou et al., 2024; Li et al., 2025a) although its use as a mediator will require adaptation to industrial-scale installations operating in continuous modes. Other recent study (Detman-Ignatowska et al., 2026b), demonstrated that coconut copra biochar significantly improved bio-H_2_ yield from sugar-beet molasses, which correlated with an increased abundance of bio-H_2_-producing bacterial taxa and copies of the *hydA* gene encoding hydrogenase I. However, this enhancement was only temporary. A gradual loss of biochar functionality was observed, leading to a decline in its stimulatory effect on bio-H₂ production and requiring periodic replacement of the biochar as well as reinoculation of the bioreactor.

## 3 Acetogenesis - different metabolic pathways, the same final products

**Figure 3:**
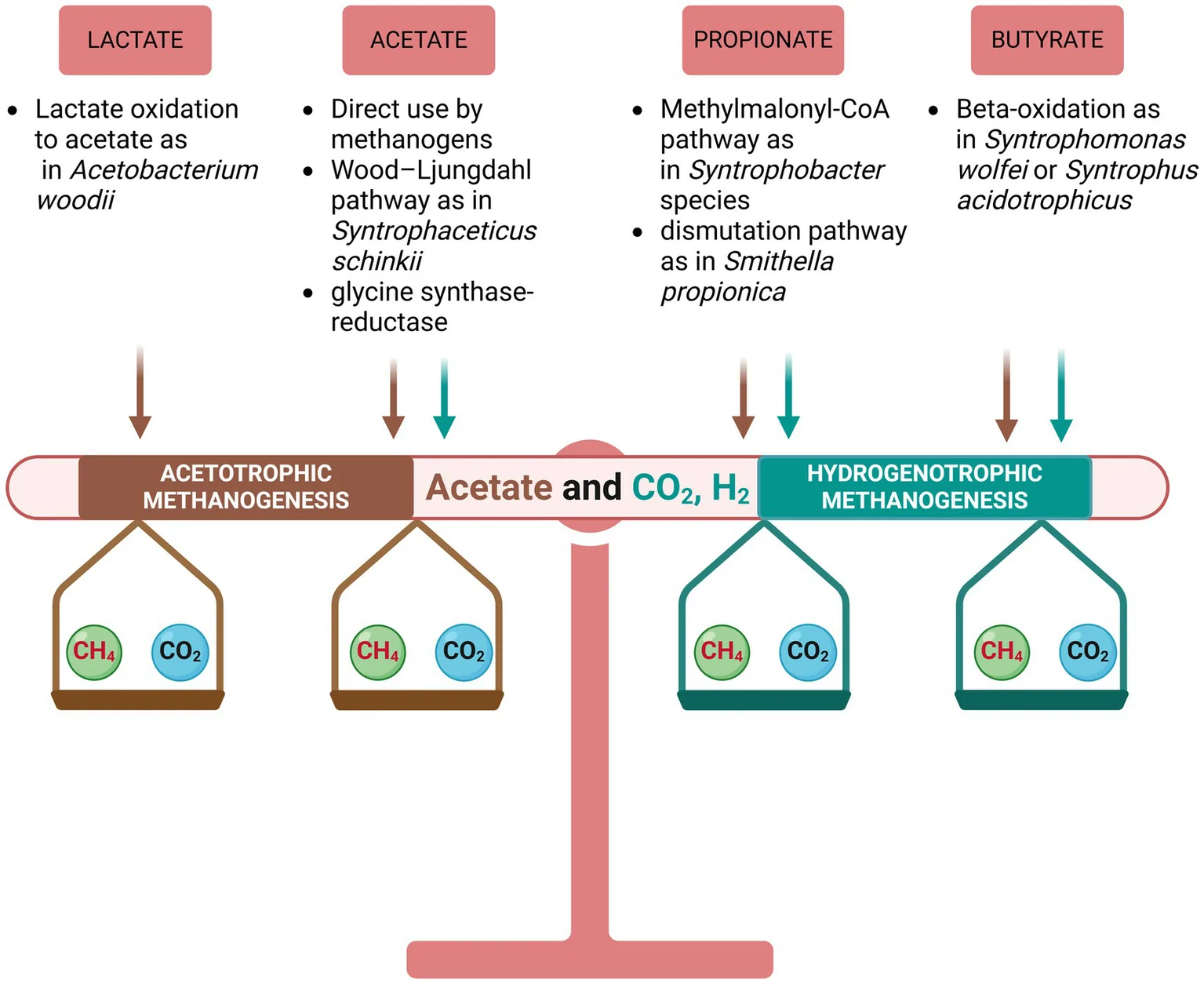
Metabolic pathways of acetogenesis and methanogenesis for the most common products of acidogenesis in bio-CH₄-yielding microbial communities. The balanced scales indicate that the bio-CH₄ production yield is comparable among all acidogenic products undergoing conversion to methane due to the involvement of different substrate-specific metabolic pathways ultimately leading to biogas formation.

**Figure 4:**
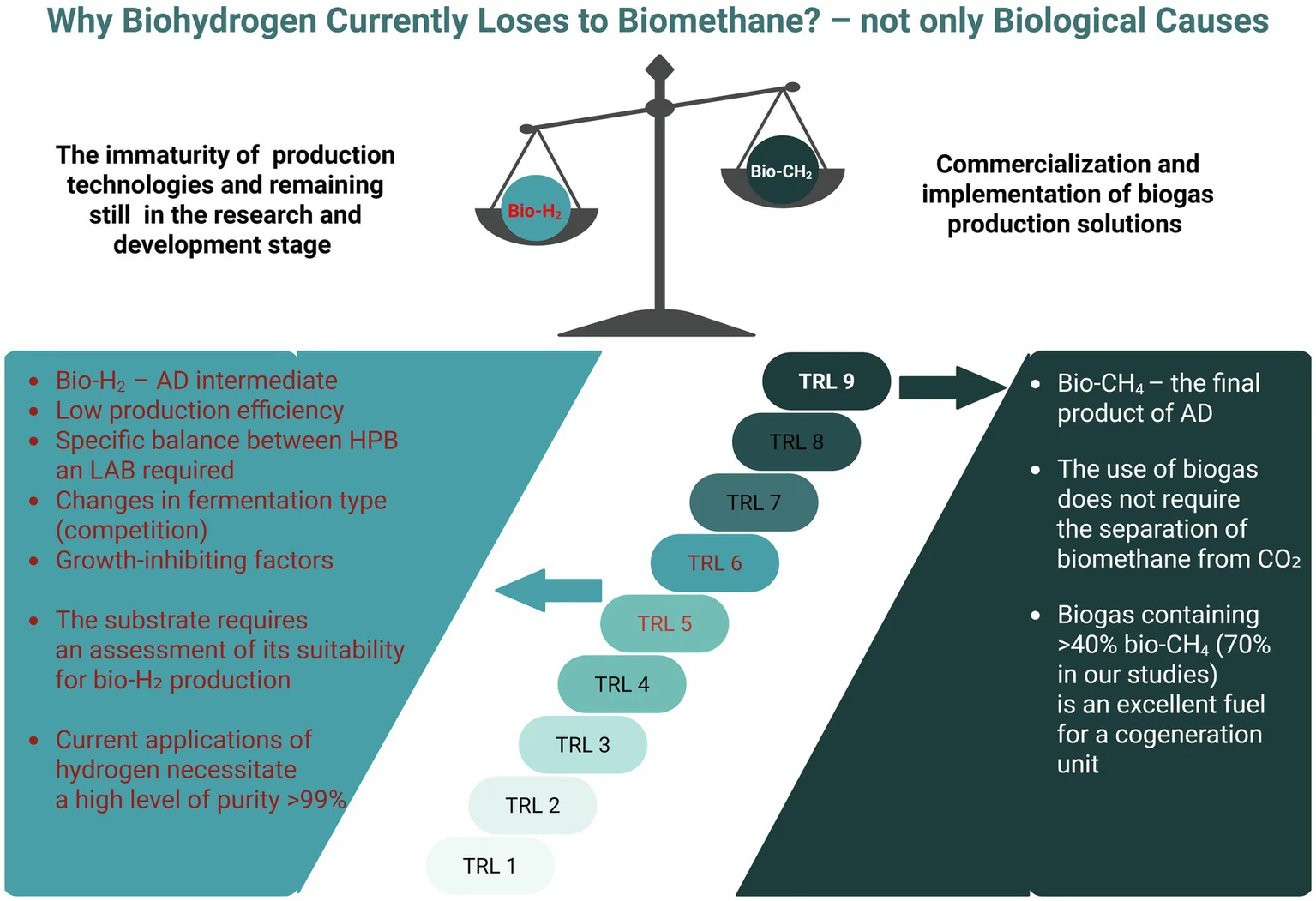
Visual summary of the challenges and limitations of bio-H₂ production compared to bio-CH4 production. The balance highlights the obstacles associated with the production of each gas.

In contrast to acidogenesis, all metabolic transformations during acetogenesis ultimately lead to the formation of substrates for methanogens (Figures 3, 4). An extensive body of literature describes the metabolic conversion of acidic fermentation products during acetogenesis and methanogenesis (Liu and Whitman, 2008; Stams and Plugge, 2009; Sieber et al., 2012; Schink and Stams, 2013). Here, we focus on the metabolic pathways involved in the transformation of the major acidogenesis products, namely lactate, acetate, butyrate, propionate, and ethanol, which ultimately lead to bio-CH_4_ formation during biogas production. In an experimental approach in which methanogenic sludge was fed with artificial media containing the dominant fermentation products lactate, acetate, propionate and butyrate (Detman et al., 2021a) the efficiency of bio-CH_4_ production was comparable in each case, although different metabolic pathways were activated. As evidenced by stable carbon isotope analysis, lactate and acetate were utilized by the acetotrophic pathway, whereas butyrate and propionate fed the hydrogenotrophic pathway of bio-CH_4_ synthesis. The dominance of the acetotrophic pathway of bio-CH_4_ production in lactate-fed bioreactors was confirmed by metagenomics and metatrancriptomic analyses (Detman et al., 2021a; Ghiotto et al., 2024). It resulted from the oxidation of lactate to acetate as described for *A. woodii* (Weghoff et al., 2015), starting with the reaction catalyzed by the FAD-dependent LDH and EtfAB. Genes encoding the enzymatic machinery for lactate utilization in *A. woodii* are widespread across the domain *Bacteria* (Detman et al., 2018b, 2021a)*.* Multi-omic analyses revealed that uncultivated *Mesotoga* species played a significant role in lactate oxidation. Interestingly, the acetoclastic archaeon *Methanothrix soehngenii* is probably capable of metabolizing lactate, as suggested by the expression of the *glcD* gene encoding a FAD-dependent LDH (Ghiotto et al., 2024). Two other pathways for anaerobic lactate oxidation are known: (i) oxidation to acetate coupled with sulfate reduction by archeons (genus *Archaeoglobus*) and bacteria belonging to *Desulfovibrio*, (ii) oxidation to acetate, CO_2_ and hydrogen in the absence of sulfate by *Desulfovibrio* coupled with syntrophic cooperation with methanogens (Stams and Plugge, 2009; Sieber et al., 2012; Schink and Stams, 2013). Butyrate is degraded *via* beta-oxidation pathway by acetogenic bacteria such as *Syntrophomonas wolfei* (Müller et al., 2009; Schmidt et al., 2013) and *Syntrophus acidotrophicus* (McInerney et al., 2007) in syntrophy with methanogenic archaea (Stams and Plugge, 2009; Sieber et al., 2012; Schink and Stams, 2013). The *β*-oxidation pathway was confirmed by metatranscriptomic approach in butyrate-fed bioreactors, with members of the *Syntrophomonadaceae* playing a central role in the degradation of this substrate (Ghiotto et al., 2024). Two main pathways of propionate degradation are recognized: (i) methylo malonyl –CoA pathway observed in *Syntrophobacter* species (Westerholm et al., 2022) and (ii) the dismutation pathway found in *Smithella propionica* (De Bok et al., 2001). In the paper of Ghiotto et al. (2024) degradation of propionate-rich medium occurred *via* both the methylmalonyl-CoA and dismutation pathways. Oxidation processes proceeded in syntrophy with the hydrogenotrophic methanogen *Methanoculleus* sp. It is notable that, contrary to the common assumption, propionate did not inhibit bio-CH_4_ production under the studied conditions.

Acetate is one of the key intermediates in AD. It can be directly converted into bio-CH_4_ and CO_2_ by acetoclastic methanogens or oxidized *via* alternative microbial pathways, including the reverse Wood–Ljungdahl (WL) pathway reported for *Syntrophaceticus schinkii* (Thauer et al., 2008; Stams and Plugge, 2009; Westerholm et al., 2010; Sieber et al., 2012; Schink and Stams, 2013) and the glycine synthase reductase (GSR) pathway proposed for *Clostridium drakei* (Song et al., 2020). These metabolic routes were also confirmed by metatranscriptomic analysis of methane-yielding microbial community fed with acetate-rich medium. Acetate was directly utilized by the acetotrophic methanogen *Methanothrix* supported by syntrophic acetate-oxidizing bacteria, primarily *Smithellaceae* sp., through the reverse WL pathway and *Thermovirgaceae* sp. through the GSR pathway (Ghiotto et al., 2024).

Ethanol can be converted via two main pathways: (i) direct oxidation to acetate via acetaldehyde, coupled with CO₂ and NADH production, as described for *Acetobacterium woodii*, without the need for syntrophic cooperation (Bertsch et al., 2016); and (ii) syntrophic oxidation to acetate, as described for *Pelobacter* spp., in association with hydrogenotrophic methanogens (Sieber et al., 2012; Schmidt et al., 2014). All these metabolic pathways generate the principal substrates required for methane production by methanogenic archaea, namely H_2_, CO_2_, and acetate. At this stage of anaerobic digestion, bio-H_2_ recovery is not feasible, as the oxidation reactions are thermodynamically unfavorable and depend on tightly coupled syntrophic interactions between bacteria and methanogens (Stams and Plugge, 2009; Sieber et al., 2012; Schink and Stams, 2013).

## 4 Alternative methods for the utilization of non-gaseous acidogenesis products

Since acidogenesis is a stage of AD, the conversion of non-gaseous acidogenic products into biogas reflects a process that naturally occurs in anaerobic ecosystems. Nevertheless, alternative strategies for the valorization of these products have been proposed, such as photofermentation and Microbial Electrolysis Cells (MECs), which enhance bio-H_2_ recovery.

Photofermentation is a form of photosynthesis carried out under anaerobic conditions by purple non-sulfur bacteria. Unlike photosynthesis in plants, bacterial photosynthesis under anaerobic conditions follows a different metabolic pathway. Instead of water, organic compounds, primarily organic acids and ethanol, serve as the source of hydrogen and electrons for carbon dioxide fixation. Consequently, oxygen is not produced during bacterial photosynthesis. Bio-H_2_ is generated during the reduction of molecular nitrogen through the activity of the enzyme nitrogenase, which can also reduce protons to bio-H₂. The main limitations of photofermentation arise from the need to maintain specific operating conditions, including adequate illumination, anaerobic conditions, and a substrate rich in organic acids but low in nitrogen content (Sağır and Hallenbeck, 2019; Li et al., 2020; Niño-Navarro et al., 2020; Policastro et al., 2022).

Microbial Electrolysis Cells (MECs) are bioelectrochemical systems in which electroactive microorganisms oxidize organic compounds, generating electrons that, with the addition of a small external voltage, can be used to produce hydrogen at the cathode. It is worth emphasizing that MEC technology can produce bio-H_2_-rich gas streams, often containing more than 905% H₂ under optimized operating conditions (Bora et al., 2022; Marchetti et al., 2025).

## 5 Why bio-H_2_ currently loses to bio-CH_4_ – bottlenecks and prospective mitigation strategies

### 5.1 Biological limitations resulting from the metabolic pathways of AD

Biogas production *via* AD is a fully commercialized technology with numerous industrial-scale installations operating worldwide (Technology Readiness Level, TRL9), whereas bio-H_2_ production technologies, including DF and other solutions such as photofermentation or MECs, remain at the laboratory, pilot or demonstration stage (TRL3 – TRL6) (Agyekum and Odoi-Yorke, 2024; Zhang et al., 2024a; Zhao et al., 2024).

In the context of DF, the biological limitations and other challenges discussed above (Figure 4) explain the current immaturity of bio-H₂ production technologies. Since many of these limitations cannot be eliminated, future development should focus on the intelligent integration of process design, feedstock selection, and stage-specific optimization. This approach may enable economically viable multi-stage AD systems in which bio-H₂, bio-CH₄, and nutrient-rich biofertilizers are co-produced and jointly contribute to the overall performance of the technology.

**Figure 5:**
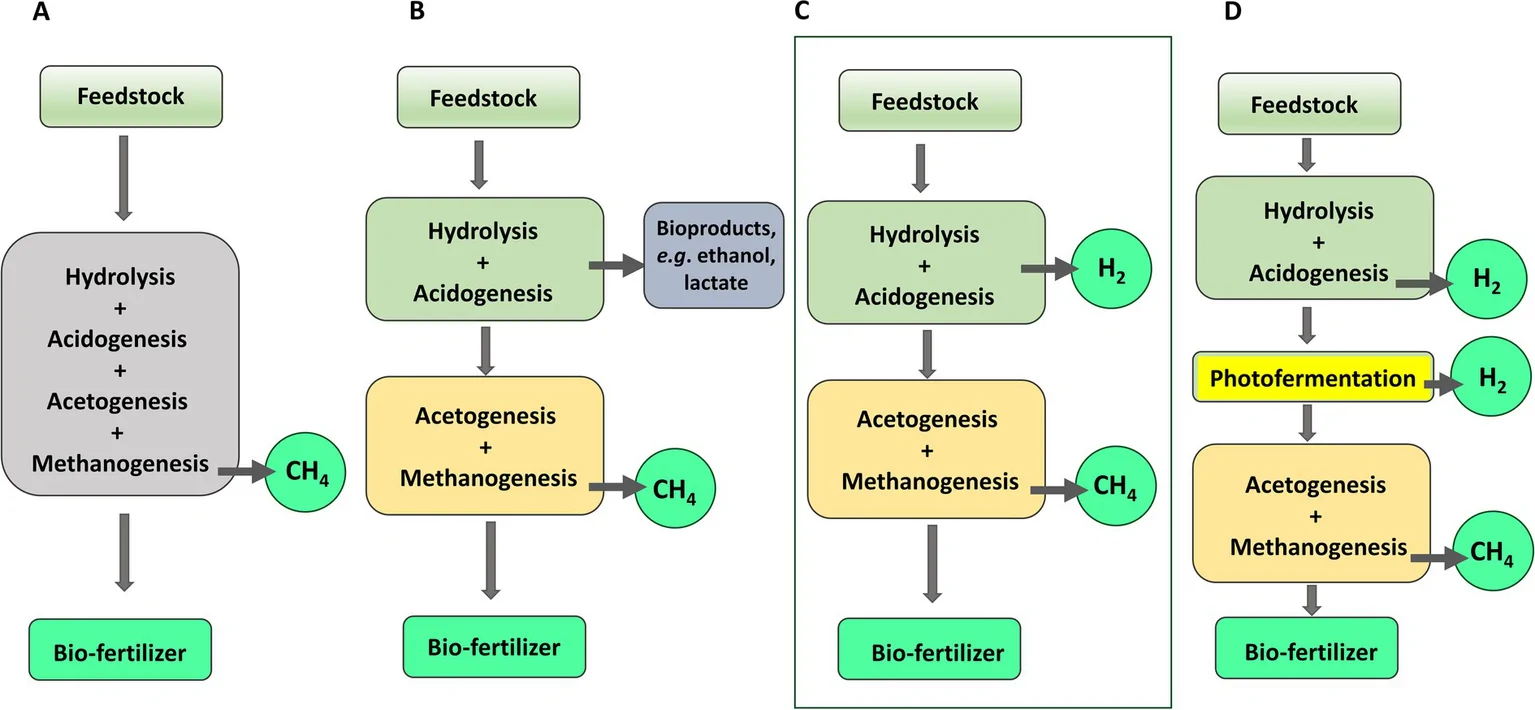
Scheme of possible configurations of integrated multiproduct anaerobic fermentation systems: **(A)** direct biogas production; **(B)** a multi-stage system in which various bioproducts can be recovered; **(C)** a multi-stage system with bio-H₂ recovery; **(D)** AD integrated with other processes, here including photofermentation between the acidogenic and methanogenic stages to increase bio-H₂ yield.

Paradoxically, thermodynamic analyses based on simple substrates such as glucose (Section 1.3) strengthen the argument for integrated multiproduct AD systems. They clearly show that bio-H_2_ is produced only through specific metabolic pathways during the acidogenesis stage, whereas other acidogenic pathways compete with bio-H_2_ production by diverting carbon and reducing equivalents toward alternative metabolites. In contrast, all acetogenic pathways generate substrates that are subsequently utilized during methanogenesis, ultimately leading to biogas production. This illustrates the unequal potential of different AD metabolic pathways with respect to bio-H_2_ and bio-CH_4_ generation.

It should be emphasized that numerous studies have examined various aspects of bio-H_2_ production during DF, including factors that inhibit the process (Ntaikou et al., 2010; Bharathiraja et al., 2016; Castelló et al., 2020; D’ Silva et al., 2023; Ahmad et al., 2024; Eggers et al., 2024; Fuess, 2024; Gbiete et al., 2024; Gupta et al., 2024; Jain et al., 2024; Devens et al., 2025; Sun et al., 2025; Özmıhçı et al., 2025). The number of published studies is so large that specific methodologies have been applied to review their findings, e.g., (D’ Silva et al., 2023; Illuminati et al., 2025).

This paper does not aim to provide another comprehensive review of the extensive literature on the topic. Instead, it focuses on metabolic pathways that are particularly relevant to multiproduct AD-based systems, in which bio-H_2_ constitutes one of several co-products.

#### 5.2 The necessity of substrate evaluation

Most review articles on DF list the various substrates used for bio-H_2_ production and evaluate their potential under different operating conditions (Tapia-Venegas et al., 2015; Bharathiraja et al., 2016; Castelló et al., 2020; D’ Silva et al., 2023; Albuquerque et al., 2024; Gbiete et al., 2024; Jain et al., 2024; Lou et al., 2024; Goren et al., 2025; Illuminati et al., 2025; Sun et al., 2025). However, it should not be assumed that bio-H_2_ can be efficiently produced from every substrate. While almost any organic matter can be converted into biogas (bio-CH_4_ and CO₂) through AD, not all substrates generate bio-H_2_ during the acidogenesis phase. Biogas containing bio-CH_4_ is the final product of AD regardless of the dominant type of acid fermentation, whereas bio-H_2_ production depends on the type of acid fermentation.

A systematic evaluation of feedstocks should be undertaken to identify the most appropriate and technically justified management pathway. Depending on substrate characteristics, this may involve direct biogas production as the simplest option (Figure 5A), a multi-stage approach in which other products (e.g., bioethanol) are first generated and the residual biomass is subsequently converted into biogas (Figure 5B), or an integrated system combining biogas production with bio-H₂ recovery during the acidogenic stage of AD (Figure 5C). Such assessments should be conducted in both laboratory-scale and pilot-scale installations operating under continuous conditions. Since process stability and long-term performance are critical for practical implementation, the potential for bio-H₂ production should be evaluated primarily in continuous-mode systems. Once stable and efficient operation has been demonstrated, the next step should be scale-up and validation under conditions representative of industrial practice (Tapia-Venegas et al., 2015; Detman et al., 2017; Eggers et al., 2024; Zhang et al., 2024b).

The importance of feedstock assessment and the need for a systematic selection of management pathways can be illustrated by the variety of by-products (e.g., molasses and sugar beet pulp) and waste streams (e.g., wash water and water from the hydraulic transport of sugar beets) generated during sugar beet processing. The wash waters represent an excellent feedstock for biogas production (Zielenkiewicz et al., 2026). Although they contain a low concentration of sucrose (equivalent to approximately 1 g L^−1^ of molasses), which is insufficient to achieve efficient bio-H_2_ production, they are sufficiently homogeneous substrates to yield biogas containing up to 80% bio-CH_4_ *via* AD. In contrast, during sugar beet pulp digestion, lactic acid and alcoholic fermentation pathways tend to dominate (Berłowska et al., 2016; Hamley-Bennett et al., 2016; Berlowska et al., 2017; Joanna et al., 2018; Alves De Oliveira et al., 2020; Usmani et al., 2022). This may be attributed to the fact that sugar beet pulp contains LAB and ascomycetous yeasts originating from the sugar beets and soil. These microorganisms are even more prevalent in wash waters. While LAB are commonly recognized as inhibitors of DF (see Section 2.3), yeasts present in these systems are overlooked, despite their potentially strong inhibitory effects on bio-H_2_ production through similar mechanisms (Detman et al., 2018a; Mielecki et al., 2024). Inhibition of bio-H_2_-producing microorganisms by metabolites excreted by other microbial populations is recognized as a significant challenge (Bundhoo and Mohee, 2016; Elbeshbishy et al., 2017; Detman et al., 2018a; Castelló et al., 2020). Moreover, ethanol, the primary product of yeast fermentation, is an attractive substrate for methanogenic consortia (Thauer et al., 2008; Stams and Plugge, 2009; Sieber et al., 2012; Schink and Stams, 2013).

#### 5.3 Electron transfer within DF bioreactors

The theoretical maximum efficiency of bio-H_2_ production *via* DF is low (33%), but the recovery of both bio-H_2_ and bio-CH_4_ through AD is still more attractive than bio-CH_4_ alone.

Current research efforts are focused on achieving bio-H₂ yields that are closer to the theoretical maximum values. Future-oriented strategies aimed at enhancing the efficiency and stability of bio-H₂ production should concentrate on facilitating extracellular electron transfer (EET) within DF microbial communities (Cheng et al., 2020; Bu et al., 2021; Cardeña et al., 2024; Lou et al., 2024; Lv et al., 2024; Tian et al., 2024). EET stimulates redox reactions involving electron bifurcation mechanisms, including the reaction catalyzed by NFOR, as well as key steps in the lactate and acetate-to-butyrate transformation pathway catalyzed by EtfAB complexes. EET enhances NADH/NAD^+^ turnover and promotes the redirection of electron flux toward hydrogenases, thereby increasing bio-H₂ production (Cardeña et al., 2024; Tian et al., 2024).

Strategies facilitating EET within DF bioreactors include the addition of conductive materials such as biochar, nickel and iron nanoparticles, magnetite partiles (Sunyoto et al., 2016; Sun et al., 2019; Cheng et al., 2020; Bu et al., 2021; Lou et al., 2024; Tian et al., 2024; Yang et al., 2024; Li et al., 2025b; Liu et al., 2025; Ramprakash and Incharoensakdi, 2025; Mishra et al., 2026) as well as the application of electro-fermentation systems (Cardeña et al., 2024; Lv et al., 2024). In electro-fermentation, increased bio-H₂ production is often accompanied by reduced biomass formation (Cardeña et al., 2024). Metagenomic analyses of DF bacterial consortia show that factors enabling EET lead to an increased abundance of bacteria from the genus *Clostridium,* as well as an enhanced metabolic potential toward bio-H_2_ production. This is reflected in a higher prevalence of genes encoding hydrogenases, PFOR, ferredoxin, hydrogenase maturation proteins, electron transfer intermediates, and enzymes related to iron metabolism (Liu et al., 2025; Detman-Ignatowska et al., 2026b; Mishra et al., 2026).

To maintain efficient and stable bio-H₂ production systems, strict control of oxidation–reduction potential and the intracellular NADH/NAD^+^ ratio, as well as facilitation of NADH/ferredoxin-mediated electron transfer, are essential.

It is noteworthy that the application of biochar-type additives must be optimized for long-term, continuous bioreactor operation. Their physicochemical properties must not deteriorate or become depleted over time (Detman-Ignatowska et al., 2026b), so their durability and sustained functionality are critical.

#### 5.4 DF bioreactors and operational conditions

DF bioreactors and operational conditions are a frequent subject of review articles (Albuquerque et al., 2024; Jain et al., 2024; Goren et al., 2025; Sun et al., 2025). To support the stable growth of bio-H₂-producing bacteria in long-term continuous systems, it is necessary to properly define the technical parameters and operational conditions of both the bioreactor and the entire installation. In bioreactors’ design and construction, several parameters are particularly important: the ratio of working volume to total volume, the height-to-diameter ratio, construction materials, the nature and characteristics of the packing material in packed bed reactors (PBR) or bioreactor mixing system in continuous stirred-tank reactor (CSTR), systems enabling effective removal of excess bacterial biomass, and procedures that stimulate EET. Since EET promotes redox reactions involving electron bifurcation mechanisms (Cardeña et al., 2024; Tian et al., 2024), it may be a key factor in maintaining the delicate balance between bio-H₂-producing bacteria and LAB discussed in Section 2.3. Long-term inoculum selection, appropriate hydraulic retention time (HRT), temperature control, pH regulation, and substrate concentration are additional factors that must be carefully refined and optimized within a comprehensive critical evaluation of each substrate discussed in Section 5.2.

#### 5.5 Purity requirements for biohydrogen and biomethane

Biogas can be directly used in cogeneration systems without separating bio-CH₄ from CO₂, although the removal of sulfur compounds, siloxanes and moisture is required (Zielenkiewicz et al., 2026). In contrast, current energy and industrial applications of H₂ require high-purity gas, whereas the bio-H₂ content of raw fermentation gas is at most about 50%. Its concentration can be increased to 60–70% by passing through a water scrubber as shown by Cieciura-Włoch et al. (2020) (Cieciura-Włoch et al., 2020). Nevertheless, this level is still insufficient for most industrial applications. Methods currently considered for separating bio-H₂ from gas mixtures include pressure swing adsorption (PSA), temperature swing adsorption (TSA), membrane separation and cryogenic separation (Tapia-Venegas et al., 2015; D’ Silva et al., 2023). However, the cryogenic method is relatively energy- and capital-intensive. This process, primarily applied in biogas upgrading, relies on the selective removal of CO₂ *via* cooling and phase transition (condensation or desublimation), taking advantage of its significantly higher phase-change temperature compared to H₂. As a result, CO₂ can be recovered in liquid or solid form (dry ice) (Xu et al., 2012; Nachtmann et al., 2015). Provided that sufficient purity is achieved and local market demand exists, dry ice may represent a value-added co-product of the process.

The ideal solution would be the direct utilization of the entire bio-H_2_-rich fermentation gas stream without the need for bio-H_2_ purification. In this context, flameless combustion represents a promising approach to achieve efficient and low-emission utilization of dilute low-calorific gas mixtures (Czyzewski et al., 2024).

#### 5.6 Comprehensive valorization of the final digestate after the AD process

In addition to biogas, the AD process also generates a residual fraction in the form of the final digestate from the methanogenic stage, which can be used as an organic fertilizer. The agricultural use of digestate is not a novelty (Zapata-Morales and Moreno-Andrade, 2025). However, increasing evidence indicates that digestate quality is strongly influenced by the process configuration (Parra-Orobio et al., 2021; Detman-Ignatowska et al., 2026a). The digestate obtained from a two-stage AD system exhibits distinct and improved properties as a fertilizer compared to that produced in conventional systems, where all four stages of AD occur within a single reactor. Chemical and microbiological analyses have demonstrated that two-stage digestate is more stable and agronomically mature. It is characterized by lower COD, reduced accumulation of fermentation intermediates, a higher pH, and higher concentrations of plant-available mineral nitrogen (ammonium and nitrate). It also contains elements essential for plant development and indole-derived auxin precursors. From a microbiological perspective, two-stage digestates are enriched in (i) bacteria involved in nitrogen metabolism and (ii) plant growth-promoting bacteria, such as *Alcaligenes* and *Liquorilactobacillus* (Detman-Ignatowska et al., 2026a). Before agricultural application, digestate must undergo ecotoxicological assessment to evaluate its potential impact on soil ecosystems and to determine whether further treatment is required to ensure environmental safety. The quality of the final digestate is highly dependent on the quality of the substrate used. Consequently, this aspect should also be considered during substrate selection and evaluation, discussed in Section 5.2.

In summary, AD should not be viewed solely as a method for biogas production, but rather as a set of microbiological processes that can serve as a platform for the development of innovative, multiproduct technologies. One of the valuable products is bio-H₂, generated during DF (the acidogenic stage of AD). However, for bio-H₂ production to move beyond the level of developmental research, it must become an integrated component of a well-designed technological value chain. Furthermore, AD can be integrated with photofermentation between the acidogenic and methanogenic stages (Figure 5D), bioelectrochemical systems such as microbial electrolysis cells (MECs), power-to-gas concepts, and carbon capture and utilization (CCU) strategies. Such hybrid approaches can improve overall process efficiency, resource recovery, and economic viability. These innovative solutions are fully consistent with the principles of sustainable development and the circular economy.

## Data Availability

The original contributions presented in the study are included in the article/supplementary material, further inquiries can be directed to the corresponding author.

## References

[ref1] Agyekum E. B. Odoi-Yorke F. (2024). Review of over two decades of research on dark and photo fermentation for biohydrogen production – a combination of traditional, systematic, and bibliometric approaches. Int. J. Hydrog. Energy 91, 1149–1169. doi: 10.1016/j.ijhydene.2024.10.218

[ref2] Ahmad A. K R. Hasan S. W. Show P. L. Banat F. (2024). Biohydrogen production through dark fermentation: recent trends and advances in transition to a circular bioeconomy. Int. J. Hydrog. Energy 52, 335–357. doi: 10.1016/j.ijhydene.2023.05.161

[ref3] Albuquerque M. M. Sartor G. D. B. Martinez-Burgos W. J. Scapini T. Edwiges T. Soccol C. R. . (2024). Biohydrogen produced via dark fermentation: a review. Methane 3, 500–532. doi: 10.3390/methane3030029 PMC12902988 41696350

[ref4] Alves De Oliveira R. Schneider R. Hoss Lunelli B. Vaz Rossell C. E. Maciel Filho R. Venus J. (2020). A simple biorefinery concept to produce 2G-lactic acid from sugar beet pulp (SBP): a high-value target approach to valorize a waste stream. Molecules 25:2113. doi: 10.3390/molecules25092113, 32365990 PMC7248869

[ref5] Atilano-Camino M. M. Luévano-Montaño C. D. García-González A. Olivo-Alanis D. S. Álvarez-Valencia L. H. García-Reyes R. B. (2020). Evaluation of dissolved and immobilized redox mediators on dark fermentation: driving to hydrogen or solventogenic pathway. Bioresour. Technol. 317:123981. doi: 10.1016/j.biortech.2020.123981, 32799081

[ref6] Baghchehsaraee B. Nakhla G. Karamanev D. Margaritis A. (2009). Effect of extrinsic lactic acid on fermentative hydrogen production. Int. J. Hydrog. Energy 34, 2573–2579. doi: 10.1016/j.ijhydene.2009.01.010

[ref7] Berlowska J. Pielech-Przybylska K. Balcerek M. Cieciura W. Borowski S. Kregiel D. (2017). Integrated bioethanol fermentation/anaerobic digestion for valorization of sugar beet pulp. Energies 10:1255. doi: 10.3390/en10091255

[ref8] Berłowska J. Pielech-Przybylska K. Balcerek M. Dziekońska-Kubczak U. Patelski P. Dziugan P. . (2016). Simultaneous Saccharification and fermentation of sugar beet pulp for efficient bioethanol production. Biomed. Res. Int. 2016, 1–10. doi: 10.1155/2016/3154929, 27722169 PMC5046097

[ref9] Bertasini D. Battista F. Mancini R. Frison N. Bolzonella D. (2024). Hydrogen and methane production through two stage anaerobic digestion of straw residues. Environ. Res. 247:118101. doi: 10.1016/j.envres.2024.118101, 38220080

[ref10] Bertsch J. Siemund A. L. Kremp F. Müller V. (2016). A novel route for ethanol oxidation in the acetogenic bacterium Acetobacterium woodii: the acetaldehyde/ethanol dehydrogenase pathway. Environ. Microbiol. 18, 2913–2922. doi: 10.1111/1462-2920.13082, 26472176

[ref11] Bharathiraja B. Sudharsanaa T. Bharghavi A. Jayamuthunagai J. Praveenkumar R. (2016). Biohydrogen and biogas – an overview on feedstocks and enhancement process. Fuel 185, 810–828. doi: 10.1016/j.fuel.2016.08.030

[ref12] Bora A. Mohanrasu K. Angelin Swetha T. Ananthi V. Sindhu R. Chi N. T. L. . (2022). Microbial electrolysis cell (MEC): reactor configurations, recent advances and strategies in biohydrogen production. Fuel 328:125269. doi: 10.1016/j.fuel.2022.125269

[ref13] Bu J. Wei H.-L. Wang Y.-T. Cheng J.-R. Zhu M.-J. (2021). Biochar boosts dark fermentative H2 production from sugarcane bagasse by selective enrichment/colonization of functional bacteria and enhancing extracellular electron transfer. Water Res. 202:117440. doi: 10.1016/j.watres.2021.117440, 34304072

[ref14] Bundhoo M. A. Z. Mohee R. (2016). Inhibition of dark fermentative bio-hydrogen production: a review. Int. J. Hydrog. Energy 41, 6713–6733. doi: 10.1016/j.ijhydene.2016.03.057

[ref15] Cardeña R. Valencia-Ojeda C. Chazaro-Ruiz L. F. Razo-Flores E. (2024). Regulation of the dark fermentation products by electro-fermentation in reactors without membrane. Int. J. Hydrog. Energy 49, 107–116. doi: 10.1016/j.ijhydene.2023.06.253

[ref16] Castelló E. Nunes Ferraz-Junior A. D. Andreani C. Anzola-Rojas M. D. P. Borzacconi L. Buitrón G. . (2020). Stability problems in the hydrogen production by dark fermentation: possible causes and solutions. Renew. Sust. Energ. Rev. 119:109602. doi: 10.1016/j.rser.2019.109602

[ref17] Centurion V. B. Rossi A. Orellana E. Ghiotto G. Kakuk B. Morlino M. S. . (2024). A unified compendium of prokaryotic and viral genomes from over 300 anaerobic digestion microbiomes. Environmental Microbiome 19:1. doi: 10.1186/s40793-023-00545-2, 38167520 PMC10762816

[ref18] Cha M. Park M. Kim S. (2025). Biological routes for biohydrogen production: a clean and carbon-free fuel. Biotechnol. J. 20:e70074. doi: 10.1002/biot.70074, 40726050 PMC12304615

[ref19] Cheng J. Li H. Ding L. Zhou J. Song W. Li Y.-Y. . (2020). Improving hydrogen and methane co-generation in cascading dark fermentation and anaerobic digestion: the effect of magnetite nanoparticles on microbial electron transfer and syntrophism. Chem. Eng. J. 397:125394. doi: 10.1016/j.cej.2020.125394

[ref20] Chojnacka A. Błaszczyk M. K. Szczęsny P. Nowak K. Sumińska M. Tomczyk-Żak K. . (2011). Comparative analysis of hydrogen-producing bacterial biofilms and granular sludge formed in continuous cultures of fermentative bacteria. Bioresour. Technol. 102, 10057–10064. doi: 10.1016/j.biortech.2011.08.063, 21908188

[ref21] Chojnacka A. Szczęsny P. Błaszczyk M. K. Zielenkiewicz U. Detman A. Salamon A. . (2015). Noteworthy facts about a methane-producing microbial community processing acidic effluent from sugar beet molasses fermentation. PLoS One 10:e0128008. doi: 10.1371/journal.pone.0128008, 26000448 PMC4441513

[ref22] Cieciura-Włoch W. Borowski S. Domański J. (2020). Dark fermentative hydrogen production from hydrolyzed sugar beet pulp improved by iron addition. Bioresour. Technol. 314:123713. doi: 10.1016/j.biortech.2020.123713, 32629374

[ref23] Corrêa A. G. Nadaleti W. C. De Souza E. G. Costa Dos Santos M. Thue P. S. Temple T. (2025). Enhancing biomass energy recovery in Brazil’s agro-industrial sector: innovating biogas, biomethane, and hydrogen production through peach syrup waste management. Renew. Energy 247:123029. doi: 10.1016/j.renene.2025.123029

[ref24] Czyzewski P. Slefarski R. Golebiewski M. Alnajideen M. Valera-Medina A. (2024). Experimental study of CO2/H2/NH3 influence on CH4 flameless combustion process in semi-industrial furnace. Energy 296:131014. doi: 10.1016/j.energy.2024.131014

[ref25] D’ Silva T. C. Khan S. A. Kumar S. Kumar D. Isha A. Deb S. . (2023). Biohydrogen production through dark fermentation from waste biomass: current status and future perspectives on biorefinery development. Fuel 350:128842. doi: 10.1016/j.fuel.2023.128842

[ref26] Das D. Veziroglu T. (2008). Advances in biological hydrogen production processes. Int. J. Hydrog. Energy 33, 6046–6057. doi: 10.1016/j.ijhydene.2008.07.098

[ref27] De Bok F. A. M. Stams A. J. M. Dijkema C. Boone D. R. (2001). Pathway of propionate oxidation by a syntrophic culture of Smithella propionica and Methanospirillum hungatei. Appl. Environ. Microbiol. 67, 1800–1804. doi: 10.1128/AEM.67.4.1800-1804.2001, 11282636 PMC92800

[ref28] Detman A. Bucha M. Treu L. Chojnacka A. Pleśniak Ł. Salamon A. . (2021a). Evaluation of acidogenesis products’ effect on biogas production performed with metagenomics and isotopic approaches. Biotechnol. Biofuels 14:125. doi: 10.1186/s13068-021-01968-0, 34051845 PMC8164749

[ref29] Detman A. Chojnacka A. Błaszczyk M. Kaźmierczak W. Piotrowski J. Sikora A. (2017). Biohydrogen and biomethane (biogas) production in the consecutive stages of anaerobic digestion of molasses. Pol. J. Environ. Stud. 26, 1023–1029. doi: 10.15244/pjoes/68149

[ref30] Detman A. Chojnacka A. Mielecki D. Błaszczyk M. K. Sikora A. (2018a). Inhibition of hydrogen-yielding dark fermentation by ascomycetous yeasts. Int. J. Hydrog. Energy 43, 10967–10979. doi: 10.1016/j.ijhydene.2018.05.004

[ref31] Detman A. Laubitz D. Chojnacka A. Kiela P. R. Salamon A. Barberán A. . (2021b). Dynamics of dark fermentation microbial communities in the light of lactate and butyrate production. Microbiome 9:158. doi: 10.1186/s40168-021-01105-x, 34261525 PMC8281708

[ref32] Detman A. Laubitz D. Chojnacka A. Wiktorowska-Sowa E. Piotrowski J. Salamon A. . (2021c). Dynamics and complexity of dark fermentation microbial communities producing hydrogen from sugar beet molasses in continuously operating packed bed reactors. Front. Microbiol. 11:612344. doi: 10.3389/fmicb.2020.612344, 33488554 PMC7819888

[ref33] Detman A. Mielecki D. Chojnacka A. Salamon A. Błaszczyk M. K. Sikora A. (2019). Cell factories converting lactate and acetate to butyrate: Clostridium butyricum and microbial communities from dark fermentation bioreactors. Microb. Cell Factories 18:36. doi: 10.1186/s12934-019-1085-1, 30760264 PMC6373154

[ref34] Detman A. Mielecki D. Pleśniak Ł. Bucha M. Janiga M. Matyasik I. . (2018b). Methane-yielding microbial communities processing lactate-rich substrates: a piece of the anaerobic digestion puzzle. Biotechnol. Biofuels 11:116. doi: 10.1186/s13068-018-1106-z, 29721040 PMC5910564

[ref35] Detman-Ignatowska A. Chojnacka A. Paul M. Jonczak J. Galas M. Dębiec-Andrzejewska K. . (2026). Single-stage vs. two-stage anaerobic digestion of molasses in a circular economy context: energy recovery and digestate fertilizer value. SSRN Elec. J. doi: 10.2139/ssrn.6301403

[ref36] Detman-Ignatowska A. Chojnacka A. Paul M. Schiro G. Jonczak J. Samborowska E. . (2026b). Impact of plant-derived biochars on biohydrogen production from sugar beet molasses in a continuous system: insights into the roles of microbial communities and chemical elements. Int. J. Hydrog. Energy 199:152812. doi: 10.1016/j.ijhydene.2025.152812

[ref37] Devens K. U. Ribeiro A. R. Camargo F. P. Sakamoto I. K. Varesche M. B. A. Silva E. L. (2025). Feasibility of successive hydrogen and methane production: effects of temperature and organic loads on energy potential and microbial dynamics. Int. Biodeterior. Biodegrad. 196:105955. doi: 10.1016/j.ibiod.2024.105955

[ref38] Diaz-Cruces V. F. García-Depraect O. León-Becerril E. (2020). Effect of lactate fermentation type on the biochemical methane potential of tequila vinasse. Bioenergy Res. 13, 571–580. doi: 10.1007/s12155-020-10093-z

[ref39] Diez-Gonzalez F. Russell J. B. Hunter J. B. (1995). The role of an NAD-independent lactate dehydrogenase and acetate in the utilization of lactate Byclostridium acetobutylicum strain P262. Arch. Microbiol. 164, 36–42. doi: 10.1007/BF02568732

[ref40] Duncan S. H. Louis P. Flint H. J. (2004). Lactate-utilizing Bacteria, isolated from human feces, that produce butyrate as a major fermentation product. Appl. Environ. Microbiol. 70, 5810–5817. doi: 10.1128/AEM.70.10.5810-5817.2004, 15466518 PMC522113

[ref41] Eggers N. Giebner F. Heinemann D. Wagner M. Birth-Reichert T. (2024). Improvement of substrate turnover through integrating dark fermentation into existing biogas plants. Biofuels Bioprod. Biorefin. 18, 855–864. doi: 10.1002/bbb.2658

[ref42] Elbeshbishy E. Dhar B. R. Nakhla G. Lee H.-S. (2017). A critical review on inhibition of dark biohydrogen fermentation. Renew. Sust. Energ. Rev. 79, 656–668. doi: 10.1016/j.rser.2017.05.075

[ref43] Etchebehere C. Castelló E. Wenzel J. Del Pilar Anzola-Rojas M. Borzacconi L. Buitrón G. . (2016). Microbial communities from 20 different hydrogen-producing reactors studied by 454 pyrosequencing. Appl. Microbiol. Biotechnol. 100, 3371–3384. doi: 10.1007/s00253-016-7325-y, 26825820

[ref44] Fan Y. Zhang F. He K. Yu D. Chen H. Tian D. . (2025). Functional microorganisms in hydrogen production: mechanisms and applications. Bioresour. Technol. 419:132007. doi: 10.1016/j.biortech.2024.132007, 39733810

[ref45] Fuess L. T. (2024). Fermentative biohydrogen production in sugarcane biorefineries: advances, challenges and prospects. Int. J. Hydrog. Energy 49, 532–553. doi: 10.1016/j.ijhydene.2023.10.092

[ref46] Fuess L. T. Ferraz A. D. N. Machado C. B. Zaiat M. (2018). Temporal dynamics and metabolic correlation between lactate-producing and hydrogen-producing bacteria in sugarcane vinasse dark fermentation: the key role of lactate. Bioresour. Technol. 247, 426–433. doi: 10.1016/j.biortech.2017.09.121, 28965073

[ref47] Fuess L. T. Zaiat M. Do Nascimento C. A. O. (2019). Novel insights on the versatility of biohydrogen production from sugarcane vinasse via thermophilic dark fermentation: impacts of pH-driven operating strategies on acidogenesis metabolite profiles. Bioresour. Technol. 286:121379. doi: 10.1016/j.biortech.2019.121379, 31051398

[ref48] García-Depraect O. Castro-Muñoz R. Muñoz R. Rene E. R. León-Becerril E. Valdez-Vazquez I. . (2021a). A review on the factors influencing biohydrogen production from lactate: the key to unlocking enhanced dark fermentative processes. Bioresour. Technol. 324:124595. doi: 10.1016/j.biortech.2020.124595, 33453519

[ref49] García-Depraect O. Gómez-Romero J. León-Becerril E. López-López A. (2017). A novel biohydrogen production process: co-digestion of vinasse and Nejayote as complex raw substrates using a robust inoculum. Int. J. Hydrog. Energy 42, 5820–5831. doi: 10.1016/j.ijhydene.2016.11.204

[ref50] García-Depraect O. León-Becerril E. (2018). Fermentative biohydrogen production from tequila vinasse via the lactate-acetate pathway: operational performance, kinetic analysis and microbial ecology. Fuel 234, 151–160. doi: 10.1016/j.fuel.2018.06.126

[ref51] García-Depraect O. Martínez-Mendoza L. J. Diaz I. Muñoz R. (2022). Two-stage anaerobic digestion of food waste: enhanced bioenergy production rate by steering lactate-type fermentation during hydrolysis-acidogenesis. Bioresour. Technol. 358:127358. doi: 10.1016/j.biortech.2022.127358, 35605777

[ref52] García-Depraect O. Muñoz R. Rodríguez E. Rene E. R. León-Becerril E. (2021b). Microbial ecology of a lactate-driven dark fermentation process producing hydrogen under carbohydrate-limiting conditions. Int. J. Hydrog. Energy 46, 11284–11296. doi: 10.1016/j.ijhydene.2020.08.209

[ref53] García-Depraect O. Muñoz R. Van Lier J. B. Rene E. R. Diaz-Cruces V. F. León-Becerril E. (2020). Three-stage process for tequila vinasse valorization through sequential lactate, biohydrogen and methane production. Bioresour. Technol. 307:123160. doi: 10.1016/j.biortech.2020.123160, 32222692

[ref54] García-Depraect O. Rene E. R. Diaz-Cruces V. F. León-Becerril E. (2019a). Effect of process parameters on enhanced biohydrogen production from tequila vinasse via the lactate-acetate pathway. Bioresour. Technol. 273, 618–626. doi: 10.1016/j.biortech.2018.11.056, 30497061

[ref55] García-Depraect O. Rene E. R. Gómez-Romero J. López-López A. León-Becerril E. (2019b). Enhanced biohydrogen production from the dark co-fermentation of tequila vinasse and nixtamalization wastewater: novel insights into ecological regulation by pH. Fuel 253, 159–166. doi: 10.1016/j.fuel.2019.04.147

[ref56] García-Depraect O. Valdez-Vázquez I. Rene E. R. Gómez-Romero J. López-López A. León-Becerril E. (2019c). Lactate- and acetate-based biohydrogen production through dark co-fermentation of tequila vinasse and nixtamalization wastewater: metabolic and microbial community dynamics. Bioresour. Technol. 282, 236–244. doi: 10.1016/j.biortech.2019.02.100, 30870689

[ref57] Gbiete D. Narra S. Mani Kongnine D. Narra M.-M. Nelles M. (2024). Insights into biohydrogen production through dark fermentation of food waste: substrate properties, inocula, and pretreatment strategies. Energies 17:6350. doi: 10.3390/en17246350

[ref58] Ghiotto G. Detman-Ignatowska A. Chojnacka A. Orellana E. De Bernardini N. Fraulini S. . (2024). Decipher syntrophies within C2-C4 organic acids-degrading anaerobic microbiomes: a multi-omic exploration. Chem. Eng. J. 489:151390. doi: 10.1016/j.cej.2024.151390

[ref59] Goren A. Y. Dincer I. Khalvati A. (2025). A comparative evaluation of dark fermentative bioreactor configurations for enhanced hydrogen production. Environ. Sci. Pollut. Res. 32, 2182–2209. doi: 10.1007/s11356-024-35873-4, 39777599

[ref60] Gupta S. Fernandes A. Lopes A. Grasa L. Salafranca J. (2024). Microbes and parameters influencing dark fermentation for hydrogen production. Appl. Sci. 14:10789. doi: 10.3390/app142310789

[ref61] Hallenbeck P. C. (2005). Fundamentals of the fermentative production of hydrogen. Water Sci. Technol. 52, 21–29. doi: 10.2166/wst.2005.0494, 16180405

[ref62] Hallenbeck P. C. (2009). Fermentative hydrogen production: principles, progress, and prognosis. Int. J. Hydrog. Energy 34, 7379–7389. doi: 10.1016/j.ijhydene.2008.12.080

[ref63] Hamley-Bennett C. Lye G. J. Leak D. J. (2016). Selective fractionation of sugar beet pulp for release of fermentation and chemical feedstocks; optimisation of thermo-chemical pre-treatment. Bioresour. Technol. 209, 259–264. doi: 10.1016/j.biortech.2016.02.131, 26978325

[ref64] Hung C.-H. Lee K.-S. Cheng L.-H. Huang Y.-H. Lin P.-J. Chang J.-S. (2007). Quantitative analysis of a high-rate hydrogen-producing microbial community in anaerobic agitated granular sludge bed bioreactors using glucose as substrate. Appl. Microbiol. Biotechnol. 75, 693–701. doi: 10.1007/s00253-007-0854-7, 17440720

[ref65] Illuminati F. Savio R. Pezzuolo A. Ferrari G. Marinello F. Guidolin M. . (2025). Biohydrogen production from agricultural and livestock by-products by dark fermentation: a data mining approach. Agriculture 15:2323. doi: 10.3390/agriculture15222323

[ref66] Jain R. Panwar N. L. Jain S. K. Gupta T. Agarwal C. Meena S. S. (2024). Bio-hydrogen production through dark fermentation: an overview. Biomass Conv. Bioref. 14, 12699–12724. doi: 10.1007/s13399-022-03282-7

[ref67] Jo J. Lee D. Park D. Park J. (2008). Biological hydrogen production by immobilized cells of Clostridium tyrobutyricum JM1 isolated from a food waste treatment process. Bioresour. Technol. 99, 6666–6672. doi: 10.1016/j.biortech.2007.11.067, 18248983

[ref68] Joanna B. Michal B. Piotr D. Agnieszka W. Dorota K. Izabela W. (2018). “Sugar beet pulp as a source of valuable biotechnological products,” in Advances in Biotechnology for Food Industry, (Amsterdam: Elsevier), 359–392.

[ref69] Kim T.-H. Lee Y. Chang K.-H. Hwang S.-J. (2012). Effects of initial lactic acid concentration, HRTs, and OLRs on bio-hydrogen production from lactate-type fermentation. Bioresour. Technol. 103, 136–141. doi: 10.1016/j.biortech.2011.09.093, 22071244

[ref70] Kougias P. G. Angelidaki I. (2018). Biogas and its opportunities—a review. Front. Environ. Sci. Eng. 12:14. doi: 10.1007/s11783-018-1037-8

[ref71] Kraemer J. T. Bagley D. M. (2007). Improving the yield from fermentative hydrogen production. Biotechnol. Lett. 29, 685–695. doi: 10.1007/s10529-006-9299-9, 17279447

[ref72] Lee D.-J. Show K.-Y. Su A. (2011). Dark fermentation on biohydrogen production: pure culture. Bioresour. Technol. 102, 8393–8402. doi: 10.1016/j.biortech.2011.03.041, 21511469

[ref73] Li F. Hinderberger J. Seedorf H. Zhang J. Buckel W. Thauer R. K. (2008). Coupled ferredoxin and Crotonyl coenzyme a (CoA) reduction with NADH catalyzed by the Butyryl-CoA dehydrogenase/Etf complex from Clostridium kluyveri. J. Bacteriol. 190, 843–850. doi: 10.1128/JB.01417-07, 17993531 PMC2223550

[ref74] Li W. Zhang Q. Cheng C. Xie Y. Liu M. Ren N. . (2025a). Quantitative analysis of the mechanism of biochar in alleviating product inhibition in different fermentative hydrogen production processes. Biochar 7:53. doi: 10.1007/s42773-025-00453-3

[ref75] Li W. Zhang Q. Cheng C. Xie Y. Liu M. Ren N. . (2025b). Quantitative analysis of the mechanism of biochar in alleviating product inhibition in different fermentative hydrogen production processes. Biochar 7:53. doi: 10.1007/s42773-025-00453-3

[ref76] Li Y. Zhang Z. Zhang Q. Tahir N. Jing Y. Xia C. . (2020). Enhancement of bio-hydrogen yield and pH stability in photo fermentation process using dark fermentation effluent as succedaneum. Bioresour. Technol. 297:122504. doi: 10.1016/j.biortech.2019.122504, 31813819

[ref77] Liu Z. Tang S. Ren Y. Chen P. Ma D. Si B. . (2025). Biohydrogen production from food waste using a novel rotational drum reactor integrated with milli-magnetite. Bioresour. Technol. 434:132822. doi: 10.1016/j.biortech.2025.132822, 40527424

[ref78] Liu Y. Whitman W. B. (2008). Metabolic, phylogenetic, and ecological diversity of the methanogenic Archaea. Ann. N. Y. Acad. Sci. 1125, 171–189. doi: 10.1196/annals.1419.019, 18378594

[ref79] Lou T. Yin Y. Wang J. (2024). Recent advances in effect of biochar on fermentative hydrogen production: performance and mechanisms. Int. J. Hydrog. Energy 57, 315–327. doi: 10.1016/j.ijhydene.2024.01.039

[ref80] Lv Y. Feng Q. Li X. Zhao Y. Pan H. Peng G. . (2024). Analysis of the contribution of different electron transfer pathways for hydrogen production in a bioelectrochemically assisted dark fermentation system. Int. J. Hydrog. Energy 72, 967–975. doi: 10.1016/j.ijhydene.2024.05.473

[ref81] Mao C. Feng Y. Wang X. Ren G. (2015). Review on research achievements of biogas from anaerobic digestion. Renew. Sust. Energ. Rev. 45, 540–555. doi: 10.1016/j.rser.2015.02.032

[ref82] Marchetti A. Cerrillo Moreno M. Lauri R. Zeppilli M. (2025). Optimizing hydrogen production through efficient organic matter oxidation performed by microbial electrolysis cells. PRO 13:1231. doi: 10.3390/pr13041231

[ref83] Mari A. G. Andreani C. L. Tonello T. U. Leite L. C. C. Fernandes J. R. Lopes D. D. . (2020). Biohydrogen and biomethane production from cassava wastewater in a two-stage anaerobic sequencing batch biofilm reactor. Int. J. Hydrog. Energy 45, 5165–5174. doi: 10.1016/j.ijhydene.2019.07.054

[ref84] Martínez-Mendoza L. J. Lebrero R. Muñoz R. García-Depraect O. (2022). Influence of key operational parameters on biohydrogen production from fruit and vegetable waste via lactate-driven dark fermentation. Bioresour. Technol. 364:128070. doi: 10.1016/j.biortech.2022.128070, 36202282

[ref85] Matsumoto M. Nishimura Y. (2007). Hydrogen production by fermentation using acetic acid and lactic acid. J. Biosci. Bioeng. 103, 236–241. doi: 10.1263/jbb.103.236, 17434426

[ref86] McInerney M. J. Rohlin L. Mouttaki H. Kim U. Krupp R. S. Rios-Hernandez L. . (2007). The genome of Syntrophus aciditrophicus: life at the thermodynamic limit of microbial growth. Proc. Natl. Acad. Sci. USA 104, 7600–7605. doi: 10.1073/pnas.0610456104, 17442750 PMC1863511

[ref87] Mielecki D. Detman A. Aleksandrzak-Piekarczyk T. Widomska M. Chojnacka A. Stachurska-Skrodzka A. . (2024). Unlocking the genome of the non-sourdough Kazachstania humilis MAW1: insights into inhibitory factors and phenotypic properties. Microb. Cell Factories 23:111. doi: 10.1186/s12934-024-02380-7, 38622625 PMC11017505

[ref88] Mishra P. Zhang R. Wang P. Geng Y. Li D. . (2026). Genome-resolved insights into Ni/Fe_2_O_3_ nanocatalyst-enhanced dark fermentative hydrogen production from food waste. Sustain. Energy Fuels 10, 2209–2221. doi: 10.1039/D5SE01709B

[ref89] Mostafa A. Im S. Kim J. Lim K.-H. Kim I. Kim D.-H. (2022). Electron bifurcation reactions in dark fermentation: an overview for better understanding and improvement. Bioresour. Technol. 344:126327. doi: 10.1016/j.biortech.2021.126327, 34785332

[ref90] Müller N. Schleheck D. Schink B. (2009). Involvement of NADH:acceptor oxidoreductase and butyryl coenzyme a dehydrogenase in reversed electron transport during syntrophic butyrate oxidation by Syntrophomonas wolfei. J. Bacteriol. 191, 6167–6177. doi: 10.1128/JB.01605-08, 19648244 PMC2747885

[ref91] Muñoz-Tamayo R. Laroche B. Walter É. Doré J. Duncan S. H. Flint H. J. . (2011). Kinetic modelling of lactate utilization and butyrate production by key human colonic bacterial species: modelling butyrate production by colonic bacterial species. FEMS Microbiol. Ecol. 76, 615–624. doi: 10.1111/j.1574-6941.2011.01085.x, 21388423

[ref92] Nachtmann K. Hofmann N. Paetzold J. Baum S. Pérez C. M. Falk O. . (2015). Dry ice and liquefied biomethane — two products from biogas for an energetic and economical upgrading of biogas plants. MAST 1, 1–7. doi: 10.15341/mast(2375-9402)/01.01.2015/001

[ref93] Nguyen L. N. Nguyen A. Q. Nghiem L. D. (2019). “Microbial community in anaerobic digestion system: progression in microbial ecology,” in Water and Wastewater Treatment Technologies, eds. Bui X.-T. Chiemchaisri C. Fujioka T. Varjani S. (Singapore: Springer Singapore), 331–355.

[ref94] Niño-Navarro C. Chairez I. Christen P. Canul-Chan M. García-Peña E. I. (2020). Enhanced hydrogen production by a sequential dark and photo fermentation process: effects of initial feedstock composition, dilution and microbial population. Renew. Energy 147, 924–936. doi: 10.1016/j.renene.2019.09.024

[ref95] Ntaikou I. Antonopoulou G. Lyberatos G. (2010). Biohydrogen production from biomass and wastes via dark fermentation: a review. Waste Biomass Valoriz. 1, 21–39. doi: 10.1007/s12649-009-9001-2

[ref96] Özmıhçı S. Hacıoğlu İ. Karapınar İ. Küs M. (2025). Valorization of green market waste for sequential biohydrogen and biomethane production. Biomass Conv. Bioref. 15, 20683–20695. doi: 10.1007/s13399-025-06620-7

[ref97] Palomo-Briones R. Trably E. López-Lozano N. E. Celis L. B. Méndez-Acosta H. O. Bernet N. . (2018). Hydrogen metabolic patterns driven by Clostridium-Streptococcus community shifts in a continuous stirred tank reactor. Appl. Microbiol. Biotechnol. 102, 2465–2475. doi: 10.1007/s00253-018-8737-7, 29335876

[ref98] Parra-Orobio B. A. Rotavisky-Sinisterra M. P. Pérez-Vidal A. Marmolejo-Rebellón L. F. Torres-Lozada P. (2021). Physicochemical, microbiological characterization and phytotoxicity of digestates produced on single-stage and two-stage anaerobic digestion of food waste. Sustain. Environ. Res. 31:11. doi: 10.1186/s42834-021-00085-9

[ref99] Policastro G. Giugliano M. Luongo V. Napolitano R. Fabbricino M. (2022). Enhancing photo fermentative hydrogen production using ethanol rich dark fermentation effluents. Int. J. Hydrog. Energy 47, 117–126. doi: 10.1016/j.ijhydene.2021.10.028

[ref100] Ramprakash B. Incharoensakdi A. (2025). Peanut shell activated carbon doped with nickel-iron nanoparticles as material for improving dark fermentative hydrogen production by Enterobacter aerogenes. Int. J. Hydrog. Energy 99, 579–588. doi: 10.1016/j.ijhydene.2024.12.175

[ref101] Ramprakash B. Lindblad P. Eaton-Rye J. J. Incharoensakdi A. (2022). Current strategies and future perspectives in biological hydrogen production: a review. Renew. Sust. Energ. Rev. 168:112773. doi: 10.1016/j.rser.2022.112773

[ref102] Regueira-Marcos L. García-Depraect O. Muñoz R. (2023). Elucidating the role of pH and total solids content in the co-production of biohydrogen and carboxylic acids from food waste via lactate-driven dark fermentation. Fuel 338:127238. doi: 10.1016/j.fuel.2022.127238

[ref103] Sağır E. Hallenbeck P. C. (2019). “Photofermentative hydrogen production,” in Biohydrogen, (Amsterdam: Elsevier), 141–157.

[ref104] Schievano A. Tenca A. Lonati S. Manzini E. Adani F. (2014). Can two-stage instead of one-stage anaerobic digestion really increase energy recovery from biomass? Appl. Energy 124, 335–342. doi: 10.1016/j.apenergy.2014.03.024

[ref105] Schievano A. Tenca A. Scaglia B. Merlino G. Rizzi A. Daffonchio D. . (2012). Two-stage vs single-stage thermophilic anaerobic digestion: comparison of energy production and biodegradation efficiencies. Environ. Sci. Technol. 46, 8502–8510. doi: 10.1021/es301376n, 22697786

[ref106] Schink B. Stams A. J. M. (2013). “Syntrophism among prokaryotes,” in The Prokaryotes, eds. Rosenberg E. DeLong E. F. Lory S. Stackebrandt E. Thompson F. (Berlin, Heidelberg: Springer Berlin Heidelberg), 471–493.

[ref107] Schmidt A. Frensch M. Schleheck D. Schink B. Müller N. (2014). Degradation of acetaldehyde and its precursors by Pelobacter carbinolicus and P. acetylenicus. PLoS One 9:e115902. doi: 10.1371/journal.pone.0115902, 25536080 PMC4275255

[ref108] Schmidt A. Müller N. Schink B. Schleheck D. (2013). A proteomic view at the biochemistry of syntrophic butyrate oxidation in Syntrophomonas wolfei. PLoS One 8:e56905. doi: 10.1371/journal.pone.0056905, 23468890 PMC3582634

[ref109] Sen B. Aravind J. Kanmani P. Lay C.-H. (2016). State of the art and future concept of food waste fermentation to bioenergy. Renew. Sust. Energ. Rev. 53, 547–557. doi: 10.1016/j.rser.2015.08.065

[ref110] Shen G.-J. Annous B. A. Lovitt R. W. Jain M. K. Zeikus J. G. (1996). Biochemical route and control of butyrate synthesis in Butyribacterium methylotrophicum. Appl. Microbiol. Biotechnol. 45, 355–362. doi: 10.1007/s002530050696

[ref111] Shima S. Birrell J. A. Stripp S. T. Caserta G. Lenz O. (2025). “Catalysis by hydrogenase,” in Iron-Sulfur Clusters, eds. Leimkühler S. Schwarz G. Lenz O. Einsle O. (Wiley), 489–540.

[ref112] Sieber J. R. McInerney M. J. Gunsalus R. P. (2012). Genomic insights into Syntrophy: the paradigm for anaerobic metabolic cooperation. Ann. Rev. Microbiol. 66, 429–452. doi: 10.1146/annurev-micro-090110-102844, 22803797

[ref113] Sikora A. (2022). Mikroorganizmy jako producenci biowodoru: Od badań podstawowych do prac rozwojowych z dziedziny biotechnologii. GAS 1, 18–25. doi: 10.15199/17.2022.12.4

[ref114] Sikora A. Baszczyk M. Jurkowski M. Zielenkiewicz U. (2013). “Lactic acid Bacteria in hydrogen-producing consortia: on purpose or by coincidence?” in Lactic Acid Bacteria - R & D for Food, Health and Livestock Purposes, ed. Kongo J. M. (Rijeka: InTech).

[ref115] Sikora A. Detman A. Chojnacka A. Blaszczyk M. K. (2017). “Anaerobic digestion: I. A common process ensuring energy flow and the circulation of matter in ecosystems. II. A tool for the production of gaseous biofuels,” in Fermentation Processes, ed. Jozala A. F. (Rijeka: InTech).

[ref116] Sołowski G. Konkol I. Cenian A. (2020). Production of hydrogen and methane from lignocellulose waste by fermentation. A review of chemical pretreatment for enhancing the efficiency of the digestion process. J. Clean. Prod. 267:121721. doi: 10.1016/j.jclepro.2020.121721

[ref117] Song Y. Lee J. S. Shin J. Lee G. M. Jin S. Kang S. . (2020). Functional cooperation of the glycine synthase-reductase and wood–Ljungdahl pathways for autotrophic growth of Clostridium drakei. Proc. Natl. Acad. Sci. USA 117, 7516–7523. doi: 10.1073/pnas.1912289117, 32170009 PMC7132306

[ref118] Stams A. J. M. Plugge C. M. (2009). Electron transfer in syntrophic communities of anaerobic bacteria and archaea. Nat. Rev. Microbiol. 7, 568–577. doi: 10.1038/nrmicro2166, 19609258

[ref119] Sun X. Wang Z. Wu W. Zhou S. Li J. Yan B. . (2025). Dark fermentation of biomass for enhanced hydrogen production: a review of pretreatment strategies, microbial enhancement, and process regulation. Int. J. Hydrog. Energy 175:151546. doi: 10.1016/j.ijhydene.2025.151546

[ref120] Sun Y. Yang G. Zhang J. Wen C. Sun Z. (2019). Optimization and kinetic modeling of an enhanced bio-hydrogen fermentation with the addition of synergistic biochar and nickel nanoparticle. Int. J. Energy Res. 43, 983–999. doi: 10.1002/er.4342

[ref121] Sunyoto N. M. S. Zhu M. Zhang Z. Zhang D. (2016). Effect of biochar addition on hydrogen and methane production in two-phase anaerobic digestion of aqueous carbohydrates food waste. Bioresour. Technol. 219, 29–36. doi: 10.1016/j.biortech.2016.07.089, 27474855

[ref122] Tapia-Venegas E. Ramirez-Morales J. E. Silva-Illanes F. Toledo-Alarcón J. Paillet F. Escudie R. . (2015). Biohydrogen production by dark fermentation: scaling-up and technologies integration for a sustainable system. Rev. Environ. Sci. Biotechnol. 14, 761–785. doi: 10.1007/s11157-015-9383-5

[ref123] Thauer R. K. Kaster A.-K. Seedorf H. Buckel W. Hedderich R. (2008). Methanogenic archaea: ecologically relevant differences in energy conservation. Nat. Rev. Microbiol. 6, 579–591. doi: 10.1038/nrmicro1931, 18587410

[ref124] Tian W. Tang Y. Ducey T. F. Khan E. Tsang D. C. W. (2024). Facilitating intracellular Electron bifurcation by mediating Flavin-based extracellular and transmembrane Electron transfer: a novel role of pyrogenic carbon in dark fermentation for hydrogen production. Environ. Sci. Technol. 58, 17766–17776. doi: 10.1021/acs.est.4c05994, 39315852

[ref125] Usmani Z. Sharma M. Diwan D. Tripathi M. Whale E. Jayakody L. N. . (2022). Valorization of sugar beet pulp to value-added products: a review. Bioresour. Technol. 346:126580. doi: 10.1016/j.biortech.2021.126580, 34923076

[ref126] Weghoff M. C. Bertsch J. Müller V. (2015). A novel mode of lactate metabolism in strictly anaerobic bacteria. Environ. Microbiol. 17, 670–677. doi: 10.1111/1462-2920.12493, 24762045

[ref127] Westerholm M. Calusinska M. Dolfing J. (2022). Syntrophic propionate-oxidizing bacteria in methanogenic systems. FEMS Microbiol. Rev. 46:fuab057. doi: 10.1093/femsre/fuab057, 34875063 PMC8892533

[ref128] Westerholm M. Roos S. Schnürer A. (2010). Syntrophaceticus schinkii gen. Nov., sp. nov., an anaerobic, syntrophic acetate-oxidizing bacterium isolated from a mesophilic anaerobic filter. FEMS Microbiol. Lett. 309, 100–104. doi: 10.1111/j.1574-6968.2010.02023.x 20546311

[ref129] Xu G. Li L. Yang Y. Tian L. Liu T. Zhang K. (2012). A novel CO2 cryogenic liquefaction and separation system. Energy 42, 522–529. doi: 10.1016/j.energy.2012.02.048

[ref130] Yang Y. Bu J. Tiong Y. W. Xu S. Zhang J. He Y. . (2024). Enhanced thermophilic dark fermentation of hydrogen production from food waste by Fe-modified biochar. Environ. Res. 244:117946. doi: 10.1016/j.envres.2023.117946, 38104915

[ref131] Yang G. Wang J. (2018). Various additives for improving dark fermentative hydrogen production: a review. Renew. Sust. Energ. Rev. 95, 130–146. doi: 10.1016/j.rser.2018.07.029

[ref132] Yang P. Zhang R. Mcgarvey J. Benemann J. (2007). Biohydrogen production from cheese processing wastewater by anaerobic fermentation using mixed microbial communities. Int. J. Hydrog. Energy 32, 4761–4771. doi: 10.1016/j.ijhydene.2007.07.038

[ref133] Zapata-Morales A. L. Moreno-Andrade I. (2025). Valorization of digestates from organic solid waste as fertilizers, soil improvers, and agricultural prebiotics: panorama and perspectives. 3 Biotech 15:333. doi: 10.1007/s13205-025-04507-y, 40951879 PMC12423007

[ref134] Zhang Q. Jiao Y. He C. Ruan R. Hu J. Ren J. . (2024a). Biological fermentation pilot-scale systems and evaluation for commercial viability towards sustainable biohydrogen production. Nat. Commun. 15:4539. doi: 10.1038/s41467-024-48790-4, 38806457 PMC11133433

[ref135] Zhang Q. Jiao Y. He C. Ruan R. Hu J. Ren J. . (2024b). Biological fermentation pilot-scale systems and evaluation for commercial viability towards sustainable biohydrogen production. Nat. Commun. 15:4539. doi: 10.1038/s41467-024-48790-4, 38806457 PMC11133433

[ref136] Zhao Z.-T. Ding J. Wang B.-Y. Bao M.-Y. Liu B.-F. Pang J.-W. . (2024). Advances in the biomass valorization in dark fermentation systems: a sustainable approach for biohydrogen production. Chem. Eng. J. 481:148444. doi: 10.1016/j.cej.2023.148444

[ref137] Zielenkiewicz U. Salamaga H. Walter T. Klim J. Szewczyk M. (2026). Extremely effective self-sufficient sugar factory wastewater treatment plant and its methanogenic microbial consortium. Chem. Eng. J. Adv. 25:100971. doi: 10.1016/j.ceja.2025.100971

