# Correction: Can bio-H_2_ compete with bio-CH_4_-unequal chances of anaerobic digestion metabolic pathways

**DOI:** 10.3389/fmicb.2026.1963866

**Published:** 2026-09-09

**Authors:** Frontiers Production Office

**Affiliations:** Frontiers Media SA, Lausanne, Switzerland

**Keywords:** anaerobic digestion, biohydrogen, biomethane, dark fermentation, metabolic pathways, microorganisms

The article type was erroneously given as Perspective. The correct article type is Review.

A few author corrections were not implemented.

A correction has been made to section: **1. Gaseous biofuels**, *1.3 Theoretical energy yield from gaseous biofuels produced via AD*, paragraph 2:

“The overall methanogenic reaction looks like this:

C_6_H_12_O_6_ + 2H_2_O → 2CH_4_ + 3CO_2_ + 2H_2_O

or in a simplified form:

C_6_H_12_O_6_ → 3CH_4_ + 3CO_2_”

A correction has been made to section: **1. Gaseous biofuels**, *1.3 Theoretical energy yield from gaseous biofuels produced via AD*, paragraph 3:

“When all stages proceed simultaneously, 3 moles of bio-CH_4_ are produced.”

A correction has been made to section: **5. Why bio-H**_**2**_ **currently loses to bio-CH**_**4**_ **– bottlenecks and prospective mitigation strategies**, *5.6 Comprehensive valorization of the final digestate after the AD process*, paragraph 2:

“Furthermore, AD can be integrated with photofermentation between the acidogenic and methanogenic stages (Figure 5D), bioelectrochemical systems such as microbial electrolysis cells (MECs), power-to-gas concepts, and carbon capture and utilization (CCU) strategies.”

There was a mistake in Figures 2–4 as published. The correction requested was not implemented.

The corrected Figures 2–4 appear below.

**Figure 2:**
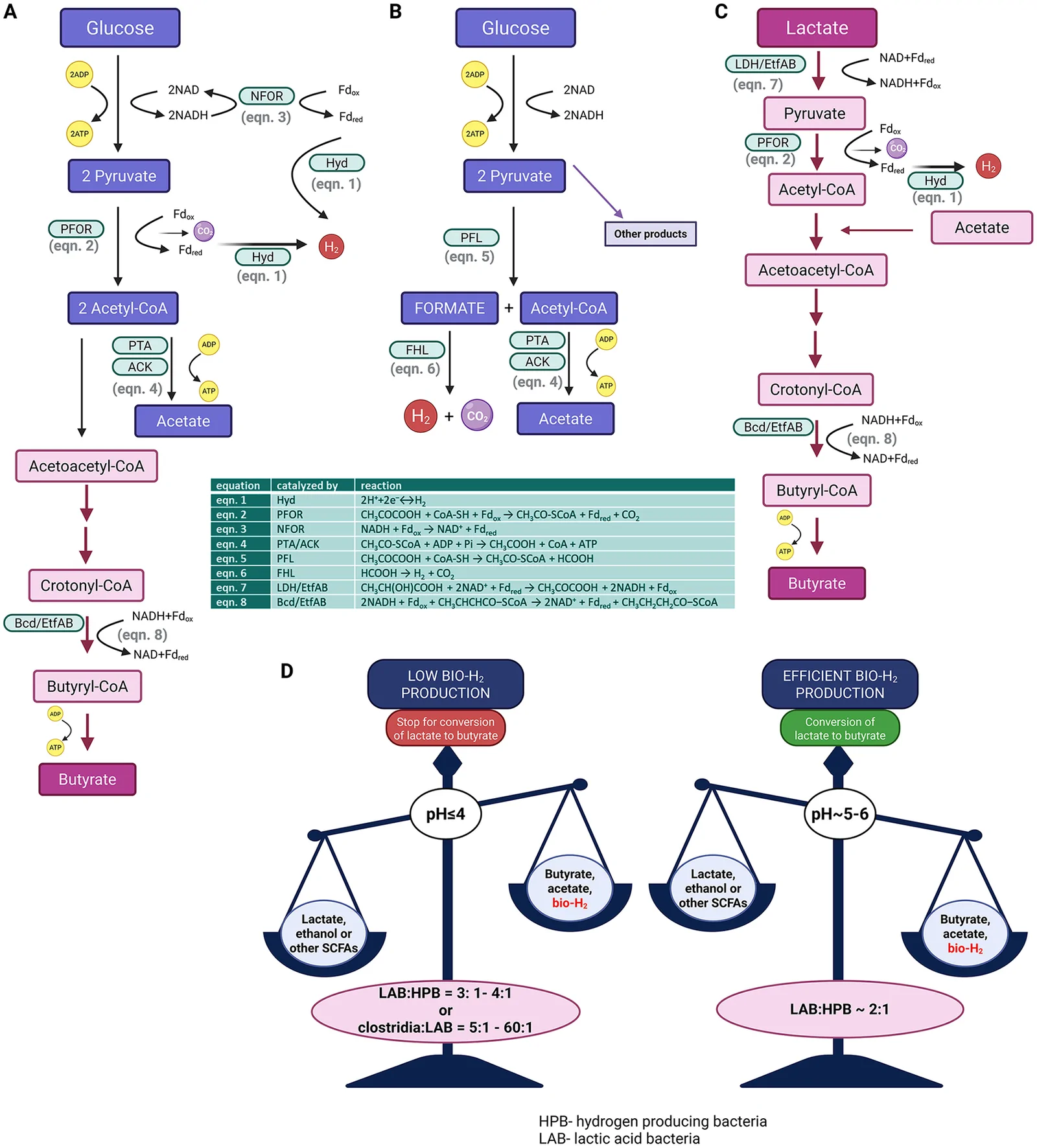
Bio-H_2_-yielding metabolic pathways in bacterial cells: **(A)** *Clostridium*-type fermentation – red arrows indicate the reactions leading to the theoretical bio-H_2_ yield of 4 moles of H_2_ per mole of glucose, according to the reaction C6H1_2_O6 + 2H_2_O → 2CH3COOH + 2CO_2_ + 4H_2_, where acetate is the only non-gaseous product. Green arrows show the pathway leading to butyrate formation, with a bio-H_2_ yield of 2 moles of H_2_ per mole of glucose, according to the reaction C6H1_2_O6 + 2H_2_O → 2H_2_ + 2CO_2_ + CH3CH_2_CH_2_COOH. **(B)** *Enterobacteriaceae*-type fermentation. **(C)** Conversion of lactate and acetate to butyrate – note the similarities to *Clostridium*-type fermentation, where butyrate is also formed. **(D)** Microbial balance in bio-H_2_-producing microbial communities based on (Detman et al., 2019, 2021c). The optimal conditions for bio-H_2_ production are determined by technical aspects and operational parameters such as hydraulic retention time (HRT), temperature, pH, feedstock composition, bioreactor construction and material, packing material, and methods of substrate feeding and distribution. Hyd, hydrogenase; PFOR, pyruvate: ferredoxin oxidoreductase; NFOR, NADH: ferredoxin oxidoreductase; PTA, phosphotransacetylase; ACK, acetate kinase; PFL, pyruvate formate lyase; FHL, formate hydrogenlyase; LDH/EtfAB, FAD-dependent lactate dehydrogenase/electron transfer flavoprotein complex; Bcd/EtfAB, butyryl-CoA dehydrogenase/EtfAB complex.

**Figure 3:**
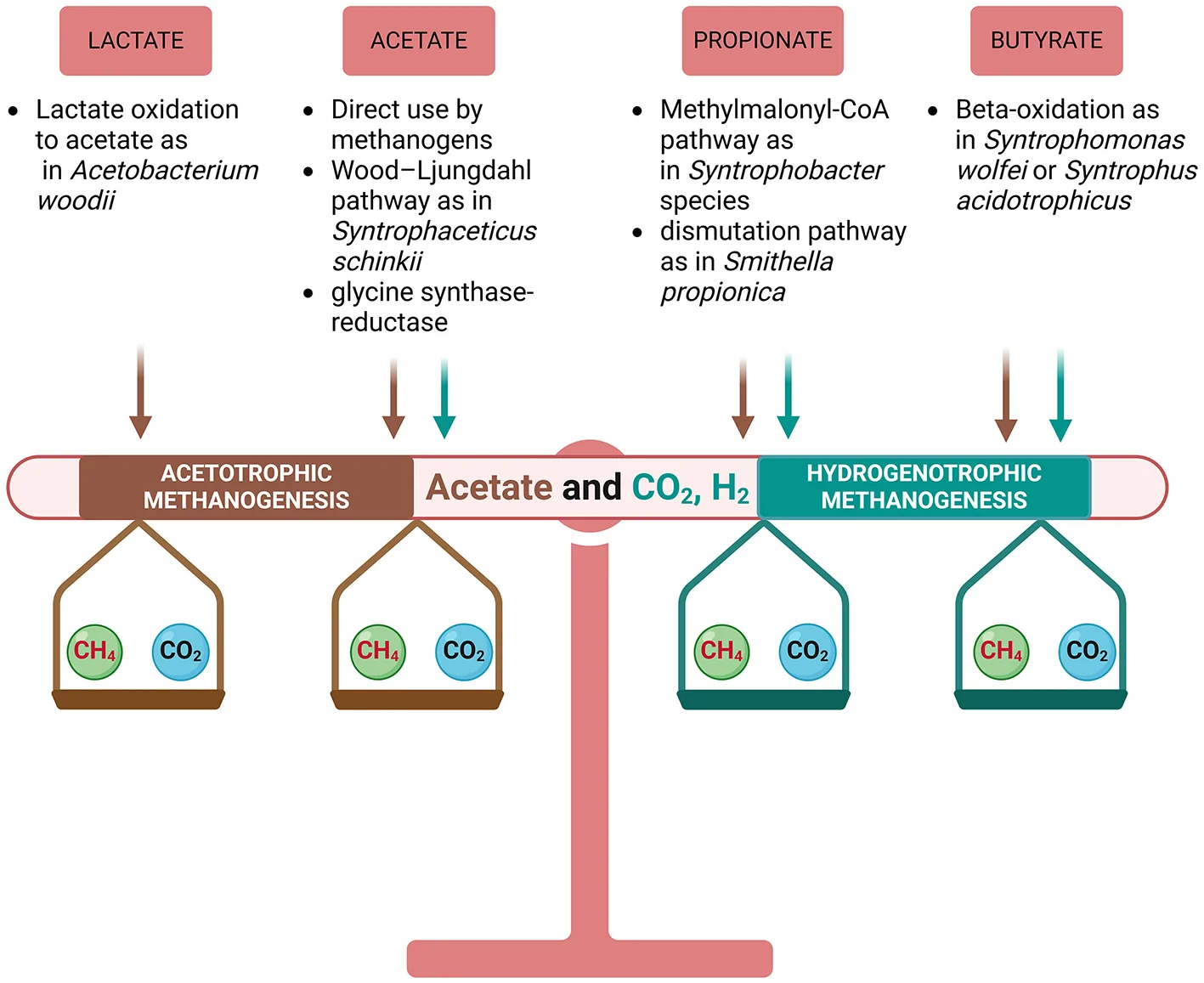
Metabolic pathways of acetogenesis and methanogenesis for the most common products of acidogenesis in bio-CH4-yielding microbial communities. The balanced scales indicate that the bio-CH4 production yield is comparable among all acidogenic products undergoing conversion to methane due to the involvement of different substrate-specific metabolic pathways ultimately leading to biogas formation.

**Figure 4:**
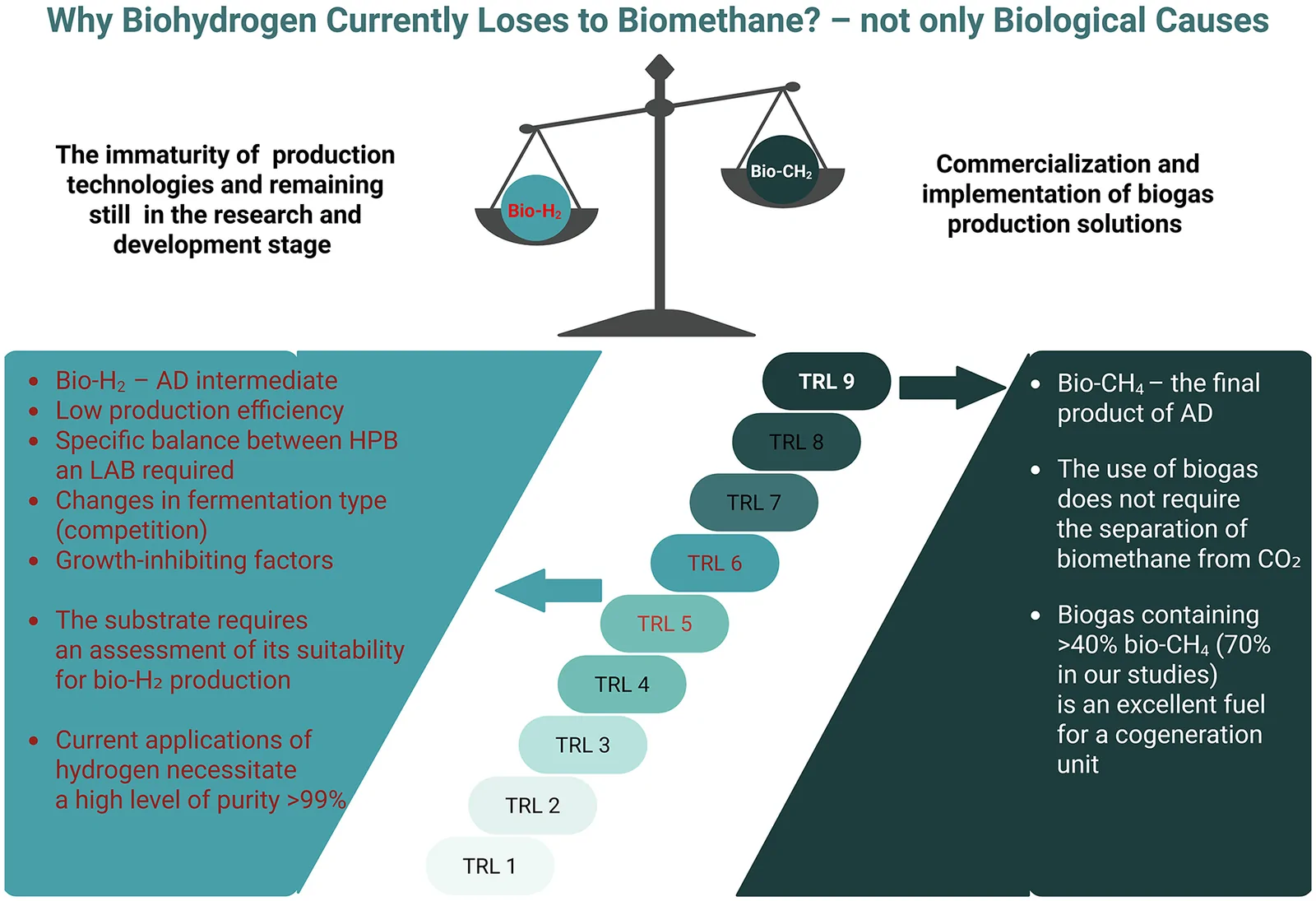
Visual summary of the challenges and limitations of bio-H2 production compared to bio-CH4 production. The balance highlights the obstacles associated with the production of each gas.

The original version of this article has been updated.

## Generative AI statement

Any alternative text (alt text) provided alongside figures in this article has been generated by Frontiers with the support of artificial intelligence and reasonable efforts have been made to ensure accuracy, including review by the authors wherever possible. If you identify any issues, please contact us.

