# Correction: Can bio-H_2_ compete with bio-CH_4_–unequal chances of anaerobic digestion metabolic pathways

**DOI:** 10.3389/fmicb.2026.2010146

**Published:** 2026-10-05

**Authors:** Frontiers Production Office

**Affiliations:** Frontiers Media SA, Lausanne, Switzerland

**Keywords:** anaerobic digestion, biohydrogen, biomethane, dark fermentation, metabolic pathways, microorganisms

The article type was erroneously given as Perspective. The correct article type is Review.

The original version of this article has been updated.

